# Late Endocrine and Metabolic Sequelae and Long-Term Monitoring of Classical Hodgkin Lymphoma and Diffuse Large B-Cell Lymphoma Survivors: A Systematic Review by the Fondazione Italiana Linfomi

**DOI:** 10.3390/cancers14061439

**Published:** 2022-03-10

**Authors:** Sergio Di Molfetta, Antonella Daniele, Chiara Gerardi, Eleonora Allocati, Carla Minoia, Giacomo Loseto, Francesco Giorgino, Attilio Guarini, Vitaliana De Sanctis

**Affiliations:** 1 Department of Emergency and Organ Transplantation, Section of Internal Medicine, Endocrinology, Andrology and Metabolic Diseases, University of Bari Aldo Moro, 70124 Bari, Italy; s.dimolfetta@libero.it (S.D.M.); francesco.giorgino@uniba.it (F.G.); 2 Experimental Oncology and Biobank Management Unit, RCCS Istituto Tumori “Giovanni Paolo II”, 70124 Bari, Italy; antonella.daniele@oncologico.bari.it; 3 Istituto di Ricerche Farmacologiche “Mario Negri” IRCCS, 20156 Milano, Italy; chiara.gerardi@marionegri.it (C.G.); eleonora.allocati@marionegri.it (E.A.); 4 Hematology Unit, IRCCS Istituto Tumori “Giovanni Paolo II”, 70124 Bari, Italy; carlaminoia@libero.it (C.M.); giacomo.loseto@gmail.com (G.L.); attilioguarini@oncologico.bari.it (A.G.); 5 Department of Medicine, Surgery and Translational Medicine, “Sapienza” University of Rome, Radiotherapy Oncology, St. Andrea Hospital, Via di Grottarossa 1035, 00189 Rome, Italy

**Keywords:** lymphoma survivors, classical Hodgkin lymphoma, diffuse large B-cell lymphoma, thyroid disease, gonadal dysfunction, osteoporosis, sarcopenia, metabolic syndrome

## Abstract

**Simple Summary:**

The aim of this systematic review, conducted by the researchers of Fondazione Italiana Linfomi (FIL), was to fill a gap in the literature regarding the follow-up of endocrine-metabolic sequelae in lymphoma patients five years and more after the end of treatments. These patients can develop a series of late toxicities, such as thyroid and gonadal dysfunctions, osteoporosis and metabolic syndrome, that contribute to comorbidity burden and could affect quality of life and overall survival. There are currently no specific indications for tailored screening programs and/or tertiary prevention for the subset of long-term lymphoma survivors treated with modern therapeutic approaches. This systematic review also aimed to understand whether there is sufficient evidence to plan modern, tailored screening programs and validated exams for the outcomes of interest based on the real incidence or prevalence of late endocrine and metabolic sequelae.

**Abstract:**

Background: Overall survival after lymphoma has improved in recent years, but the high prevalence of late treatment-related sequelae has been observed as a counterpart. Method: In this systematic review, FIL researchers aimed to: (i) estimate the incidence or prevalence of late endocrine-metabolic sequelae, (ii) evaluate the effects of modern therapeutic approaches on incidence or prevalence of late endocrine-metabolic sequelae, and (iii) determine whether there is evidence of follow-up schemes for their screening/early diagnosis in the subset of long-term classical Hodgkin lymphoma (cHL) and diffuse large B-cell lymphoma (DLBCL) survivors treated at adult age. The MEDLINE, Embase and the Cochrane Library databases were searched for relevant articles published up to October, 2020. The study selection process was conducted by three independent reviewers and was reported according to the Preferred Reporting Items for Systematic Reviews and Meta-Analyses (PRISMA) guidelines. A risk of bias assessment was performed using the Cochrane tool for randomized trials and the Newcastle-Ottawa Scale for observational studies. Results: In the final analysis, eight studies were included, four of which focused on thyroid disease, two on gonadal dysfunction, one on bone disease and one on metabolic syndrome. Hypothyroidism was reported in up to 60% of adult cHL survivors and was frequently recorded even with modern radiotherapy approaches. Menopause occurred in 52–72% of women after chemotherapy. An 86% reduction in vertebral density was reported following R-CHOP-like chemotherapy. Sarcopenia and metabolic syndrome were reported in 37.9% and 60% of patients, respectively. No validated screening protocols were found for the early diagnosis of long-term treatment-related endocrine and metabolic sequelae, thus the authors finally suggest the execution of screening exams according to the risk category which were identified in the epidemiologic studies.

## 1. Introduction

Overall survival for several types of cancer has improved progressively over recent years. Among hematological malignancies, some histotypes of lymphoma present high cure rates thanks to the availability of modern imaging techniques and more effective treatments. Lymphomas are a heterogeneous group of malignancies of the immune system that in most cases require front-line chemotherapy (CT) and/or radiotherapy (RT). Second-line treatment for classical Hodgkin lymphoma (cHL) and aggressive non-Hodgkin’s lymphoma (NHL) consists in high-dose chemotherapy and consolidation with autologous stem cell transplant (ASCT), for young and eligible patients [1,2,3,4,5,6]. According to the Italian cancer registry, the five-year survival rate in Italy for cHL was 82% for male patients and 87% for female patients while five-year survival rate was 64% and 67% for male and female NHL patients [7]. Accordingly, in Italy in 2020 there were 67,000 patients with cHL and 156,400 patients with NHL who were alive five years after diagnosis [7]. Among the different histotypes of NHL, diffuse large B-cell lymphoma (DLBCL) is the most frequent variant, constituting about 30% of new diagnoses and having a five-year cure rate of about 60% [8].

Due to the increasing number of lymphoma survivors, several long-term toxicities of diverse treatment modalities have come to the attention of hematologists and other physicians who take care of lymphoma survivors, making the follow-up multidisciplinary [9,10]. The main categories of long-term toxicity in populations of cHL and NHL patients treated in adulthood are, in order of frequency, cardiovascular diseases, secondary cancers, endocrine-metabolic, neurological sequelae and infertility [11,12,13,14,15]. Since the cumulative incidence of therapy-related disorders among lymphoma survivors does not seem to have plateaued, the burden of this ever-growing clinical issue will continue to increase in the coming years.

Endocrine-metabolic sequelae can occur both during the five years of follow-up post-treatment and beyond. The main endocrine toxicities involve the thyroid gland, the gonads and bone metabolism [11]. Metabolic syndrome (MetSyn) and progressive loss of skeletal muscle mass and function (sarcopenia) have also recently been described as long-term effects of chemotherapy and high-dose steroids, especially in NHL survivors [16,17]. In long-term follow-up, endocrine and non-endocrine disorders appear to be closely connected to each other. In fact, apart from infertility and sexual dysfunction, hypogonadism may lead to an increased risk of osteoporosis, cardiovascular disease, depression and possibly impaired cognitive function [18]. Hypothyroidism, either subclinical or overt, can also be associated in both males and females with weight gain, bradycardia, hypercholesterolemia, chronic fatigue, memory impairment, depression and hypersensitivity to cold [17]. Of note, therapy-related endocrine disorders can affect long-term survivors not only physiologically, but also psychologically. Indeed, in a registry-based cohort study, 1035 adults with cHL or NHL were investigated with the aim of assessing the prevalence of endocrine disorders and psychosocial impairment: 44% of cHL patients and 55.5% of NHL patients self-reported lack of energy, tiredness and inefficiency in doing daily activities [19]. Finally, MetSyn is associated with high cardiovascular risk due to central obesity, insulin resistance, hypertension, high plasma triglyceride levels and low plasma HDL cholesterol levels [16,17].

Historically, radiotherapy of the neck and of the pelvis have been associated with damage to the thyroid and gonadal gland. However, there has been a progressive reduction in radiation dose and volume in the last few decades, from the older extended-field radiation therapy (EFRT) to involved-field RT (IFRT) to the modern concept of involved-nodal/involved-site RT (INRT/ISRT) [20,21]. Moreover, the introduction of intensity-modulated radiation therapy (IMRT) may have contributed to changing the current clinical scenario of late radiation-related toxicities by delivering very highly conformed radiation beams to very well-defined radiation volumes. The new chemotherapy protocols and the introduction of biological therapies may have also improved the characteristics and/or incidence of thyroid, gonadal and bone toxicities when compared to older therapeutic approaches.

The present systematic review, conducted by Fondazione Italiana Linfomi (FIL) researchers, aimed to (i) estimate the incidence or prevalence of late endocrine-metabolic sequelae in cHL and DLBCL survivors treated in the adulthood, (ii) find out whether modern radiotherapy approaches have an effect on the actual incidence or prevalence of the above-mentioned sequelae, and (iii) determine whether there is any evidence of programmed schemes for the screening/early diagnosis of late endocrine-metabolic sequelae in this subset of long-term survivors treated at the adult age. The results of the bibliographic search were compared with the available guidelines to deduce indications for an appropriate and effective early detection and follow-up program for long-term lymphoma survivors.

## 2. Materials and Methods

This systematic review is part of a series of analyses exploring the management and follow-up of long-term lymphoma survivors to support FIL position statements [12,13,14,15]. The scope of the position statements, as well as the clinical questions and population/intervention/control/outcome elements (PICOs) for each question, were discussed and agreed on by the FIL Long-Term Survivor Committee and presented at the FIL congress in 2019.

We used the Preferred Reporting Items for Systematic reviews and Meta-Analyses (PRISMA) guidelines to report the results [22]. This Systematic review has not been registered.

### 2.1. Study Identification

MEDLINE (via PubMed), the Cochrane Library and EMBASE were systematically searched for documents indexed between January, 1990 and October 2020, with no language or publication type restrictions. Search terms included extensive controlled vocabulary (MeSH and EMTREE) and free-text keywords, combining the conditions (Hodgkin disease, diffuse large B-cell lymphoma), interventions (both first-line and second-line treatments) and outcomes of interest (e.g., thyroid disease, gonadal dysfunctions, bone disease, sarcopenia and metabolic syndrome). Details on the search strategies can be found in the (Supplementary Materials Files S1–S3). We checked the reference lists of relevant studies to retrieve further studies and congress abstracts and searched study registries for unpublished or ongoing studies.

### 2.2. Eligibility Criteria

We included both primary studies (randomized controlled trials, prospective and retrospective cohort studies, and registry studies) and systematic reviews including these study designs. We included studies involving long-term (≥5 years disease- or treatment-free) adult (≥18-year-old at diagnosis) cHL or DLBCL survivors. Outcome measures included incidence of long-term endocrine sequelae such as thyroid disease, gonadal dysfunctions, osteoporosis and metabolic syndrome, with emphasis on any potential effect of modern radiotherapy approaches (IMRT, dose/volume reduction). Moreover, we assessed whether early detection of long-term endocrine sequelae due to specific follow-up could provide clinical benefit.

Table 1, Table 2, Table 3 and Table 4 report the clinical questions and corresponding PICOs addressed by this systematic review. The evaluation focused on patients treated with first-therapy with/without second-line therapy including ASCT; allogeneic stem cell transplant was an exclusion criterion.

### 2.3. Study Selection and Data Extraction

Three reviewers independently screened the title and abstract of retrieved documents to select the studies for each PICO (VDS, SDM, AD). They then reviewed the corresponding full-text articles to confirm eligibility and extracted the relevant information from the included studies. A second round of review to check eligibility and data extraction to increase the accuracy of the process was done, and all discrepancies were resolved by consensus and arbitration by two other authors (CG, EA).

Data collected from each study included the following: (1) study identifier (first author, year of publication); (2) reference; (3) other publication; (4) study design; (5) population; (6) study duration; (7) follow-up; (8) sample size; (9) intervention/control group (10) outcome measure; (11) main results; (12) conclusion; (13) risk of bias/quality assessment. A standardized Excel spreadsheet (Microsoft Corporation, 2007; available at https://office.microsoft.com/excel, accessed on 27 September 2021) was used for data extraction.

### 2.4. Risk of Bias and Quality of Evidence Assessment

We assessed the methodological quality of the included systematic reviews using the AMSTAR 2 tool [23] (available at https://amstar.ca/Amstar-2.php, accessed on 27 September 2021), the risk of bias for RCTs using the Cochrane risk-of-bias (ROB) tool [24] (available at https://methods.cochrane.org/bias/resources/rob-2-revised-cochrane-risk-bias-tool-randomized-trials, accessed on 27 September 2021) and the quality of cohort and registry studies using the Newcastle-Ottawa Scale [25] (available at http://www.ohri.ca/programs/clinical_epidemiology/oxford.asp, accessed on 27 September 2021). The risk of bias and quality of evidence assessment was done by one reviewer and checked by another.

### 2.5. Data Synthesis

As we expected a substantial degree of heterogeneity among the included studies, we did not pool data in meta-analyses. For each clinical question, the included studies providing relevant information were narratively summarized and tabulated to highlight similarities and differences in their methods and results. Each specific clinical question and the corresponding PICOs were tabulated (Table 1, Table 2, Table 3 and Table 4).

## 3. Results

### 3.1. Thyroid Diseases

#### 3.1.1. Incidence and Prevalence

What is the incidence or prevalence of thyroid diseases in long-term cHL or DLBCL survivors treated with first- and/or second-line CT/RT and ASCT?

The incidence or prevalence of the following clinical diseases were investigated: abnormal thyroid function tests (overt or subclinical hypothyroidism, hyperthyroidism), autoimmune thyroid diseases (Hashimoto’s thyroiditis, Graves’ disease), thyroid nodules (single nodule, multiple nodules, thyroid cancer).

**Figure 1:**
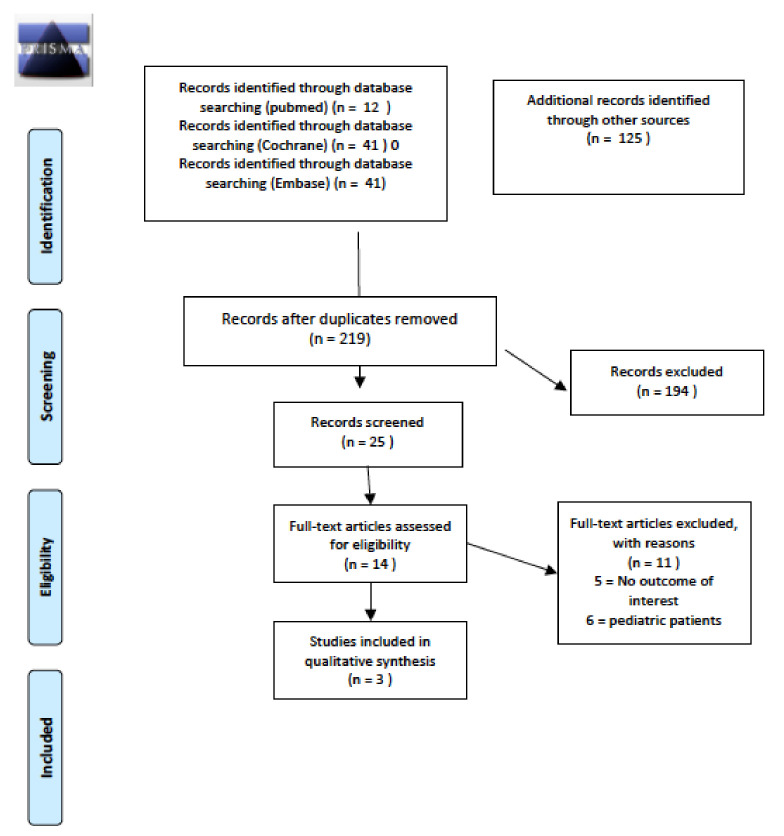
PRISMA for prevalence and/or incidence of long-term thyroid disease.

The initial literature search revealed a total of 219 articles, three of which fully matched the aim of this systematic review. Details of the whole screening process and reasons for exclusion are reported in Figure 1.

All three studies included cHL survivors who had undergone first-line treatment [26,27,28]; one was a randomized study (RCT) [26], while the other two were cohort studies [27,28].

The prospective RCT was conducted on a series of 73 cHL patients with early-stage disease and mediastinal involvement, treated at the Department of Radiation Oncology and the Hematology Department of “La Sapienza” University of Rome from 1983–1989 (median follow-up: 114 months, range 22–174 months). Patients were randomized into two groups based on their initial treatment: the first group included 37 patients treated with supradiaphragmatic radiotherapy and paraaortic irradiation (STNI), while the second group included 36 patients treated with STNI preceded by one course of Adriamycin (doxorubicin)-bleomycin-vinblastine-dacarbazine (ABVD). The occurrence of late toxicities was a secondary endpoint of the study, which was primarily aimed to assess the rates of treatment response and relapse. The authors reported two cases of hypothyroidism in the group treated with CT/RT (5.5%) [26]. No statistical evaluations were available in this study.

In the cohort study by Bethge et al., 177 long-term classical cHL survivors who had been treated with CT, RT alone, or combined CT/RT according to the protocols of the German Hodgkin Study Group were assessed between 1994 and 1997 for thyroid disease by clinical examination, thyroid function tests and ultrasound imaging, when indicated [27]. RT approaches included extended-field RT alone or in combination with CT for early-stage disease and extended-/involved-field RT plus CT for intermediate-stage disease. In advanced stage disease, RT was planned based on the presence of either initial bulky disease or residual tumor burden. Dose fractionation in 1.5–2 Gy daily administered over a 3- to 6-week period (total radiation dose 30–40 Gy) was used. The majority of patients (54%) had received cyclophosphamide-vincristine-procarbazine-prednisone (COPP)/ABVD. The median follow-up was 70 months (range 12–243) and was comparable for the subgroups CT, RT and CT/RT. Fifty-one (28%) out of 177 patients were found to have thyroid abnormalities. Hypothyroidism was diagnosed through thyroid function tests in 48 patients (27%) and was either subclinical (20%) or overt (7%). None of the patients with overt hypothyroidism had yet developed clinical symptoms, and all were then treated with thyroxin substitution before any symptoms developed. No patient treated with CT alone developed hypothyroidism, in contrast to the 15/44 (34%) patients in the RT alone group (*p* < 0.001) and the 33/98 (34%) patients in the combined CT/RT group (*p* < 0.001). The addition of CT did not increase the RT-associated risk for hypothyroidism (*p* < 0.05). Male and female patients presented the same frequency of hypothyroidism, and median age at onset was 32 years. Three patients (1.7%) were diagnosed with hyperthyroidism (nodular goiter, Graves’ disease, thyrotoxicosis factitia). The authors concluded that about one third of cHL patients who had received RT to the neck developed hypothyroidism and that the maximum of thyroid damage occurred prior to 10 years since RT. The screening protocol of thyroid dysfunction, which required that thyroid blood tests (TSH, FT3, FT4) and clinical thyroid gland examination be performed annually, made it possible to detect asymptomatic forms, for which thyroid hormone replacement therapy was initiated.

The cohort study by Illes et al. included 151 cHL survivors treated in the years 1970–2000 with CT (different schedules) and/or RT (either extended- or involved-field RT, with a total radiation dose of 30–44 Gy) [28]. Thyroid status was assessed through TSH, FT3, FT4, anti-TPO, anti-Tg and anti-TSH receptor measurement. One hundred and eleven (73.5%) subjects were euthyroid, while a diagnosis of subclinical hypothyroidism, overt hypothyroidism and hyperthyroidism was made in 26 (17.2%), 12 (8.0%) and two (1.3%) patients, respectively. Of note, thyroid antibody positivity was found in 28/151 (18.6%) patients. The statistical analysis for subgroups according to stage, general symptoms at diagnosis, age at diagnosis, age at evaluation and sex did not show any significant differences. Hypothyroidism was more frequently detected in patients who had undergone mantle or neck RT (*n* = 104) than in patients treated with CT alone (*p* = 0.005). In most cases, hypothyroidism occurred from the sixth year after RT.

According to The Newcastle-Ottawa Scale (NOS), the quality of the study by Maurizi Enrici et al. was low because, although the length of follow-up was good, the main biases were the lack of description of the diagnostic methodology for the assessment of thyroid dysfunction and the case definition, considering that one course of ABVD is not a treatment standard and could have therefore determined the low rate of hypothyroidism [26]. The quality of the study by Bethge et al. was high for all the considered items [27]. The quality of the study by Illés et al. was intermediate because, although the description of the diagnostic methodology for the assessment of thyroid dysfunction was good, the main biases were the length of follow-up and the case definition, as patients with complete remission for at least one year were also included [28].

#### 3.1.2. Treatment Comparison

Has the incidence or prevalence of thyroid diseases in long-term cHL or DLBCL survivors treated with first- and/or second-line CT/RT and ASCT changed with the introduction of modern radiotherapy?

**Figure 2:**
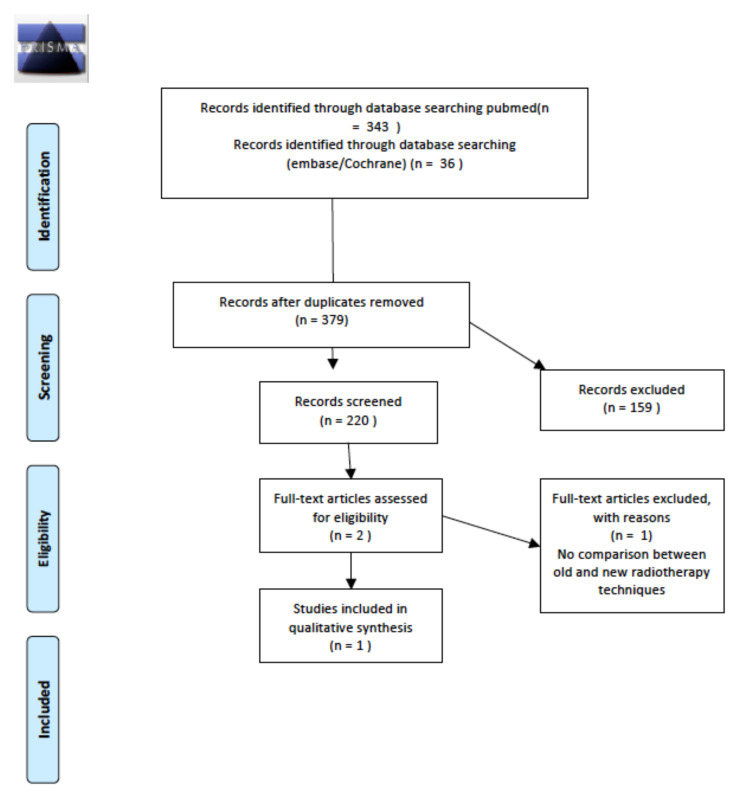
PRISMA for long-term thyroid disease after the introduction of modern radiotherapy approaches.

We analyzed the incidence or prevalence of thyroid disorders after the introduction of new RT approaches (e.g., 3DCRT, IMRT, dose/volume reduction) in comparison with previous RT regimens (e.g., 2DRT, extended-field RT), considering as outcomes the number of cases of an abnormal thyroid function test, autoimmune thyroid disease, nodular pathology of the thyroid and/or thyroid cancer. A total of 220 abstracts were screened; the two relevant abstracts were then retrieved as full texts. Of these, one was excluded, and the other was included in the final sample and relative analysis. Details of the whole screening process, including reasons for full-text exclusion, are reported in Figure 2.

The included study was a retrospective cohort study conducted from 2009 to 2014 [29]. The authors focused on the incidence of hypothyroidism among a series of 90 cHL patients with mediastinal involvement with or without neck involvement treated with ABVD, followed by ISRT delivered with the IMRT technique. Seventeen patients (19%) had received second-line CT for relapse or refractory disease. These patients were compared to a group of 50 cHL patients treated from 2001 to 2009 with ABVD, followed by IFRT delivered by 3D conformal RT (3D-CRT) with anteroposterior/posteroanterior fields. Both groups were well matched for the proportion of patients with bilateral or unilateral neck involvement. All patients had a normal pretreatment thyroid function test. Serum thyroid-stimulating hormone (TSH) and free thyroxin (FT4) were evaluated during the follow-up and considered abnormal if the value was above the normal range. After a median follow-up of 41.1 months (range 29.1–53.1 months) for the IMRT group and 32.0 months (range 18.9–45.1 months) for the 3D-CRT group, 59 patients (66%) in the IMRT group developed hypothyroidism (median time to onset 13 months, range 1.5–48.8 months), compared to 20 patients (40%) in the 3D-CRT group (median time to onset 19.5 months, range 3–82 months). Moreover, three-year rates of freedom from hypothyroidism were 56.1% for the 3D-CRT group and 40% for the IMRT group (*p* = 0.057). Small thyroid volumes and several dosimetric variables were associated with hypothyroidism, the most influential of which was the proportion of the thyroid receiving 25 Gy (V25) or the absolute volume of thyroid that was spared from 25 Gy (VS25Gy).

According to the NOS, the quality of the retrospective study by Pinnix et al. was low because, although the assessment of the outcomes and the clinical outcome of interest were well defined (the comparison between old and new radiotherapy approaches), the main biases were the length of follow-up, which was less than 60 months, and the enrollment of an uncertain number of pediatric patients (median age 28.5 years, range 14–70 years) in a series of 50 patients treated with 3D-CRT [29].

#### 3.1.3. Efficacy of Planned Follow-Up Schemes to Early Diagnosis of Thyroid Diseases in Long-Term cHL or DLBCL Survivors Treated with First- and/or Second-Line CT/RT and ASCT?

We evaluated the following follow-up schemes: scheduled follow-up/monitoring planned (e.g., annual thyroid function test, anti-thyroid autoantibody assay, ultrasound, ultrasound-guided fine needle aspiration, etc.) versus no follow-up/monitoring planned or follow-up/monitoring planned with different modalities, timing and frequencies. The outcomes considered were the incidence or prevalence of an abnormal thyroid function test, autoimmune thyroid disease, nodular thyroid disease, thyroid cancer, problems related to overdiagnosis, quality of life and mortality.

**Figure 3:**
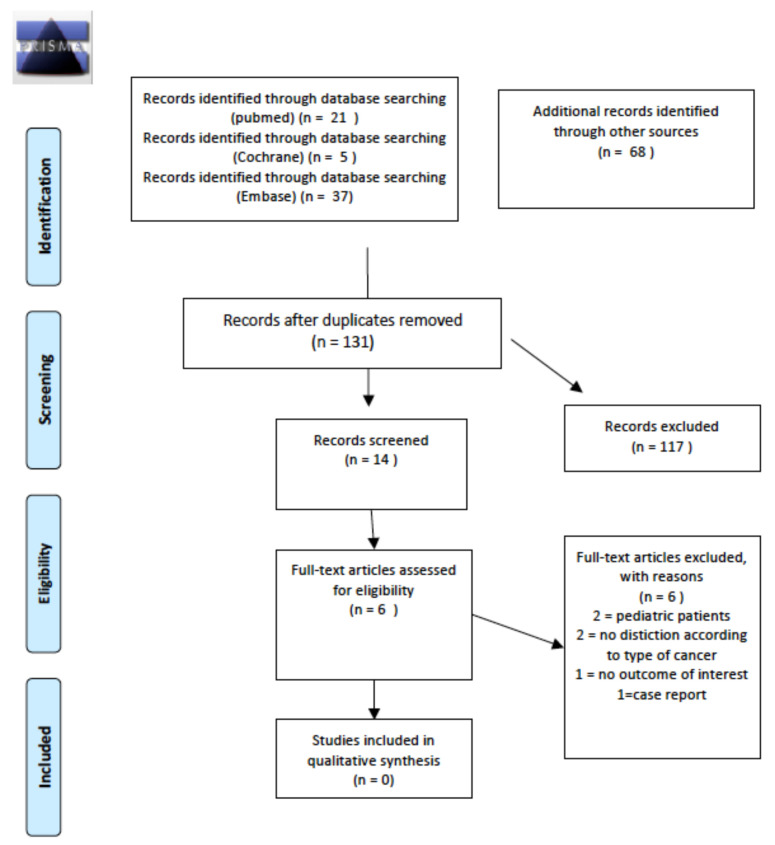
PRISMA for long term thyroid disease follow-up schemes.

A total of 14 abstracts were screened; six relevant articles were retrieved as full texts. Of these, all were excluded from the final sample and relative analysis. Details of the whole screening process, including reasons for full-text exclusion, are reported in Figure 3.

### 3.2. Gonadal Dysfunctions

#### 3.2.1. What Is the Incidence or Prevalence of Testicular Dysfunction in Long-Term cHL or DLBCL Survivors Treated with First- and Second-Line CT/RT and ASCT? What Is the Incidence or Prevalence of Ovarian Dysfunction in Long-Term cHL or DLBCL Survivors Treated with First- and/or Second-Line CT/RT and ASCT?

The literature search was based on the following clinical states: (i) incidence or prevalence of abnormal testosterone secretion (low serum testosterone and/or elevated LH levels); (ii) incidence or prevalence of symptoms of estrogen deprivation (hot flashes, vaginal dryness, dyspareunia).

**Figure 4:**
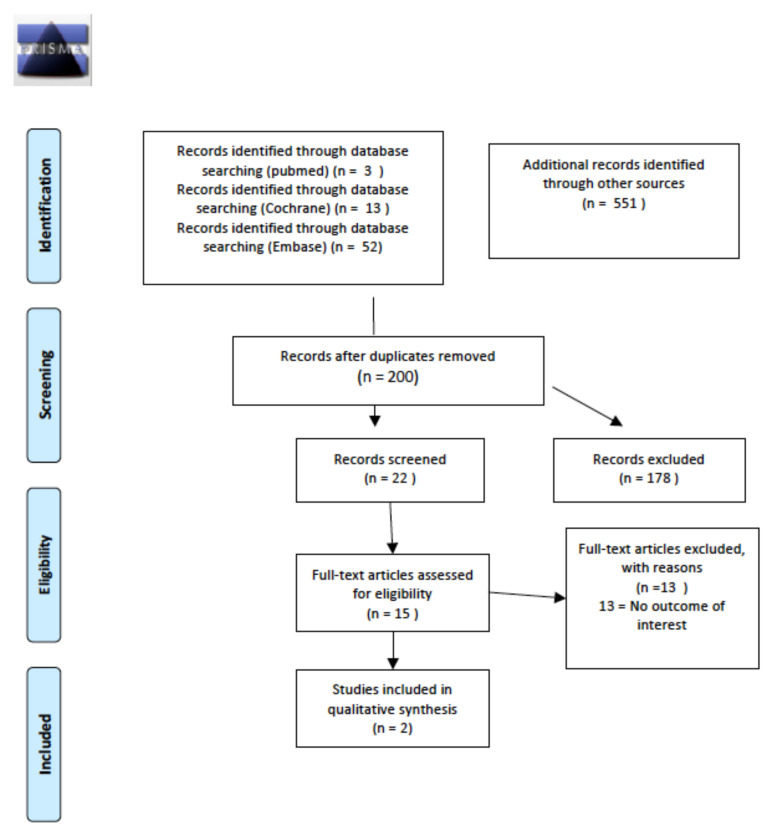
PRISMA for prevalence and/or incidence of long-term gonadal dysfunctions.

The literature search initially found 200 relevant articles, two of which were included in the final sample. Both studies were retrospective and involved long-term survivors who had undergone first-line treatment. Details of the whole screening process, including reasons for full-text exclusion, are reported in Figure 4.

In a series of patients with cHL aged <40 years at the time of treatment (RT or mustine-vinblastine-procarbazine-prednisolone [MVPP]-based CT ± mantle RT) and in first remission at the time of assessment, a remarkable incidence rate of menopause (13/18 patients, 72%) was observed up to 12 years after treatment with MVPP [30].

Recently, the Mabthera International Trial investigated the long-term impact of CHOP-like regimens on ovarian function was assessed in a cohort of 46 women with CD20-positive DLBC [31] with a median follow-up of 14 years. The menstrual status of 10 patients could not be assessed due to surgical menopause or concurrent oral contraceptives/hormone replacement therapy. Of the 36 patients not on exogenous hormone medications, 17 (48%) reported having regular menstrual bleeding and 19 (52%) being in menopause. Notably, amenorrhea had persisted after CTin 8/19 patients, whereas menopause had occurred in 11 patients after temporary recovery of regular menstrual cycles. Importantly, the total score for menopausal symptoms according to the menopause rating scale was available for 35 patients. Among these patients, 25.7%, 17.1%, 37.1% and 20% had experienced no/few, mild, moderate or severe menopausal symptoms, respectively.

According to the NOS, the quality of the retrospective study by King et al. [30] was high, while the quality of the retrospective study by Meissner et al. [31] was intermediate because several outcomes were assessed by questionnaire.

#### 3.2.2. Treatment Comparison

Has the incidence or prevalence of testicular dysfunction in long-term cHL or DLBCL survivors treated with first- and/or second-line CT/RT and ASCT changed with the introduction of modern radiotherapy? Has the incidence or prevalence of ovarian dysfunction in long-term cHL or DLBCL survivors treated with first- and/or second-line CT/RT and ASCT changed with the introduction of modern radiotherapy?

We analyzed the incidence of gonadal disorders after the introduction of new RT approaches (e.g.,3D-CRT, IMRT, dose/volume reduction) in comparison with previous RT regimens (e.g., 2D-RT, EFRT). The outcomes considered were (i) the number of cases of abnormal spermatogenesis, abnormal testosterone secretion (low serum testosterone and/or elevated LH levels); (ii) the number of cases of secondary (transient, permanent) amenorrhea; iii) the number of patients reporting symptoms of estrogen deprivation.

**Figure 5:**
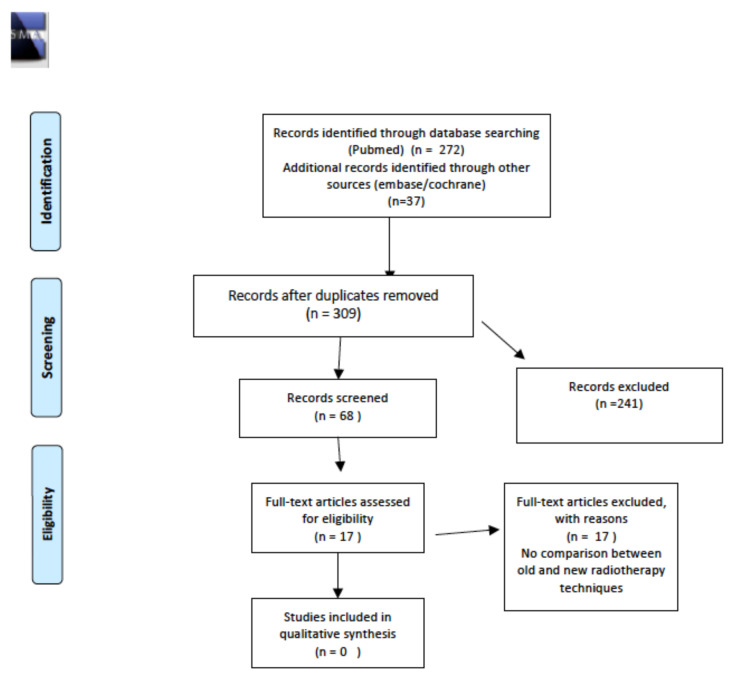
PRISMA for long-term gonadal dysfunctions after the introduction of modern radiotherapy approaches.

A total of 68 abstracts were screened, with 17 relevant publications retrieved as full texts. Of these, all were excluded from the final sample and relative analysis because no comparison between old and new radiotherapy approaches (e.g., 3D-CRT, IMRT, dose/volume reduction) was made to analyze changes in the incidence of the outcomes of interest. Details of the whole screening process, including reasons for full-text exclusion, are reported in Figure 5.

#### 3.2.3. Efficacy of Planned Follow-Up Schemes in the Diagnosis of Testicular Dysfunction in Long-Term cHL or DLBCL Survivors Treated with First- and/or Second-Line CT/RT and ASCT. Efficacy of Planned Follow-Up Schemes in the Diagnosis of Ovarian Dysfunction Long-Term cHL or DLBCL Survivors Treated with First- and/or Second-Line CT/RT and ASCT?

We evaluated the following follow-up schemes for male patients: annual total testosterone dosage, LH, etc. versus no follow-up/monitoring planned or follow-up/monitoring planned with different modalities, times and frequencies, evaluating as outcomes: alterations in testosterone secretion, problems related to overdiagnosis, quality of life and mortality. We evaluated the following follow-up planned for female patients: annual hormone test dosage (FSH, LH, estradiol, inhibin B, AMH, progesterone) and annual transvaginal pelvic ultrasound versus no follow-up/monitoring planned or follow-up/monitoring planned with different modalities, including different components, times and frequencies, considering as outcomes secondary amenorrhea, problems related to overdiagnosis, quality of life and mortality.

**Figure 6:**
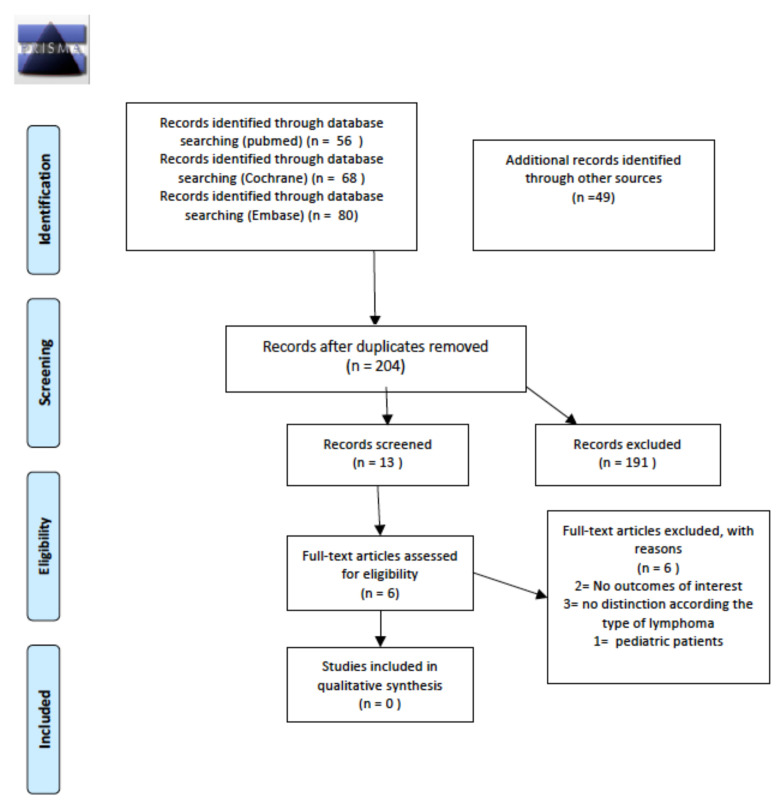
PRISMA for long term gonadal dysfunctions follow-up schemes.

A total of 13 abstracts with six relevant publications were retrieved as full texts. Of these six, all were excluded from the final sample and relative analysis. Details of the whole screening process, including reasons for full-text exclusion, are reported in Figure 6.

### 3.3. Bone Diseases

#### 3.3.1. Incidence or Prevalence

What is the Incidence or Prevalence of Changes in Bone Quality and Bone Mineral Density in Long-Term cHL or DLBCL Survivors Treated with First- and/or Second-Line CT/RT and ASCT?

**Figure 7:**
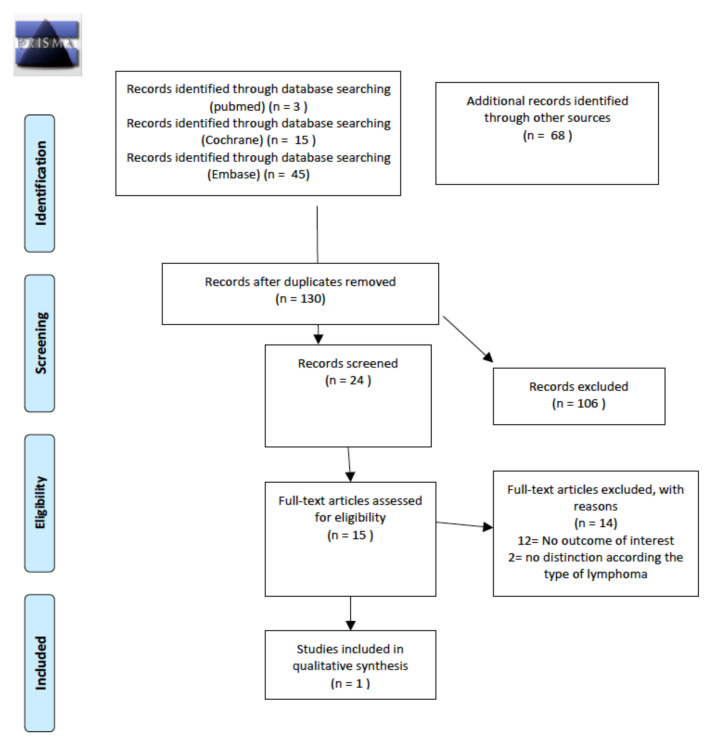
PRISMA for prevalence and/or incidence of long-term bone disease.

The following bone diseases were included as outcomes: osteopenia, osteoporosis and fractures. Out of 15 evaluated articles, only one paper was found to be relevant to our research question. Details of the whole screening process, including reasons for article exclusion during the full-text review, are reported in Figure 7.

In a single-centre retrospective study, Svedsen et al. evaluated changes from baseline in vertebral bone density (VD) in Hounsfield units (HU) at the L3 level in 111 DLBCL patients who received first-line treatment with CHOP (cyclophosphamide-doxorubicin-vincristine-prednisone) or CHOP-like chemotherapy and who underwent pre- and post-treatment vertebral computed tomography (CT) or a positron emission tomography/CT(PET/CT) scan [32]. Five percent of patients had received bisphosphonate, and 16% calcium plus vitamin D during chemotherapy. After a median follow-up of 5.2 years, post-treatment VD decreased to 86% of pretreatment value (*p* < 0.001), with neither female sex nor older age (>70 years) resulting in greater reductions of VD. Compression fractures were found in 16/111 (14%) patients. They concluded that a significant reduction in VD following R-CHOP(-like) treatment, together with the possible occurrence of vertebral fractures, made the problem clinically relevant. The use of high-dose steroids in the CHOP regimen was deemed the principal cause of a reduction in bone mineral density, and the damage is not reversible without appropriate treatment.

According to the NOS, the quality of the retrospective study by Svedsen et al. [32] was high for all the items.

#### 3.3.2. Treatment Comparison: Has the Incidence or Prevalence of Changes in Bone Quality and Mineral Density in Long-Term cHL or DLBCL Survivors Treated with First- and/or Second-Line CT/RT and ASCT Changed with the Introduction of Modern Radiotherapy?

We analyzed the incidence or prevalence of changes in bone quality and mineral density after the introduction of new RT approaches 3DCRT, IMRT, dose/volume reduction) in comparison with previous RT regimens (2DRT, extended-field RT), considering osteopenia, osteoporosis, and fractures as outcomes.

**Figure 8:**
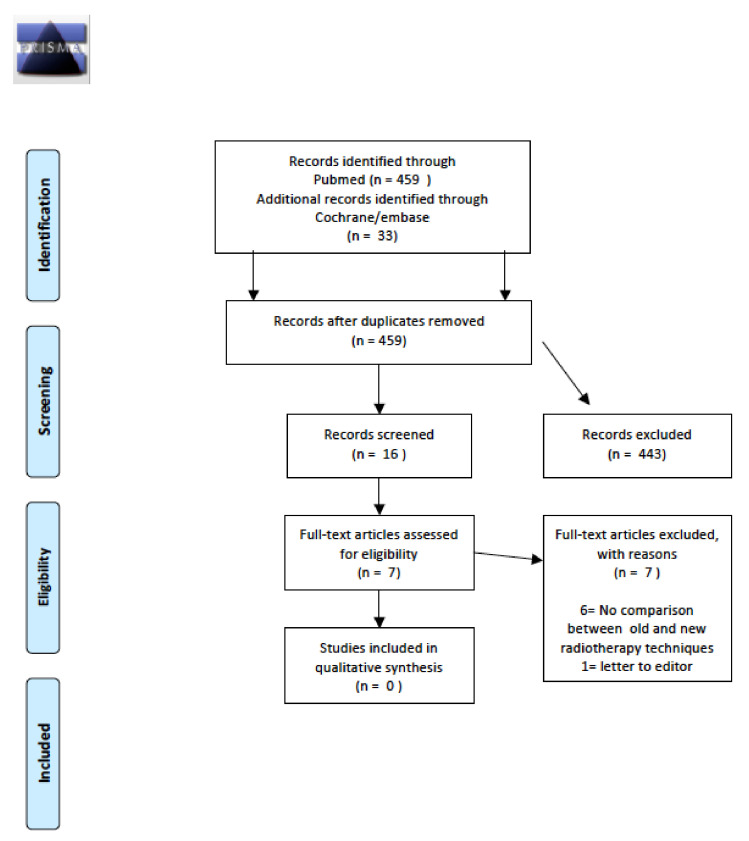
PRISMA for long-term bone disease after the introduction of modern radiotherapy approaches.

A total of 16 abstracts with seven relevant publications were retrieved as full texts. Of these, all were excluded from the final sample and relative analysis because no comparison between old and new RT approaches (3D-CRT, IMRT, dose/volume reduction) was made to analyze any changes in prevalence/incidence of the outcomes of interest. Details of the whole screening process, including reasons for full-text exclusion, are reported in Figure 8.

#### 3.3.3. Efficacy of Planned Follow-Up Schemes in Diagnosing Changes in Bone Mineral Density and Quality in Long-Term cHL or DLBCL Survivors Treated with First- and/or Second-Line CT/RT and ASCT?

We evaluated the following follow-up schemes: annual calcium and vitamin D dosage and annual bone densitometry versus no follow-up/monitoring planned or follow-up/monitoring planned with different modalities, timing and frequencies, considering as outcomes osteoporosis, osteopenia, fracture, quality of life and mortality.

**Figure 9:**
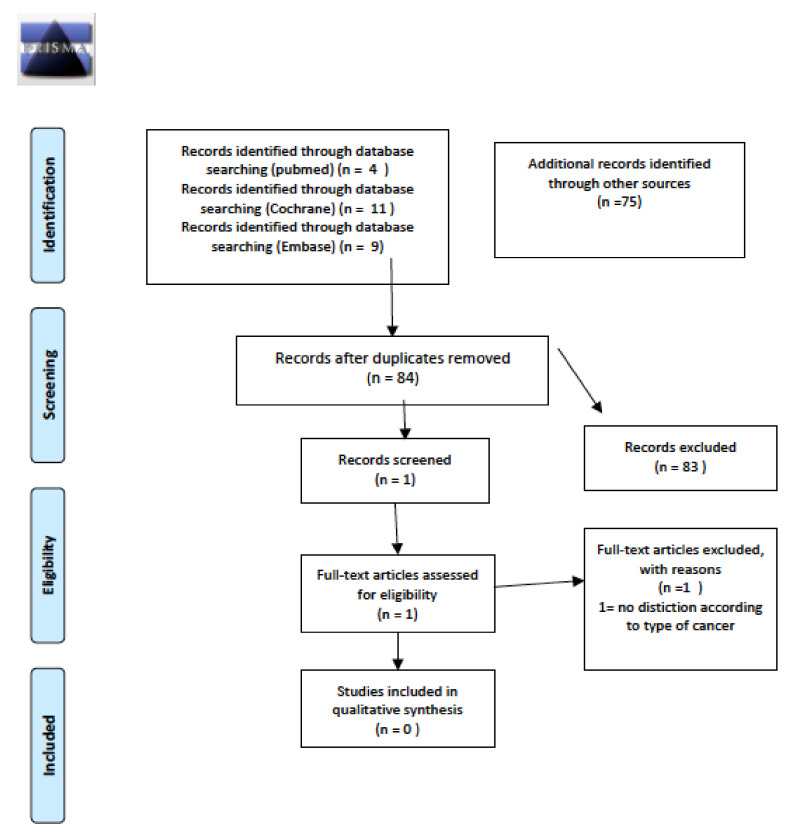
PRISMA for long-term bone disease follow-up schemes.

One abstract was screened, with one relevant publication retrieved as a full text; this one full-text article was excluded from the final sample and relative analysis. Details of the whole screening process, including reasons for full-text exclusion, are reported in Figure 9.

### 3.4. Metabolic Syndrome

#### 3.4.1. What Is the Incidence of Metabolic Syndrome in Long-Term cHL or DLBCL Survivors Treated with First- and/or Second-Line CT/RT and ASCT?

**Figure 10:**
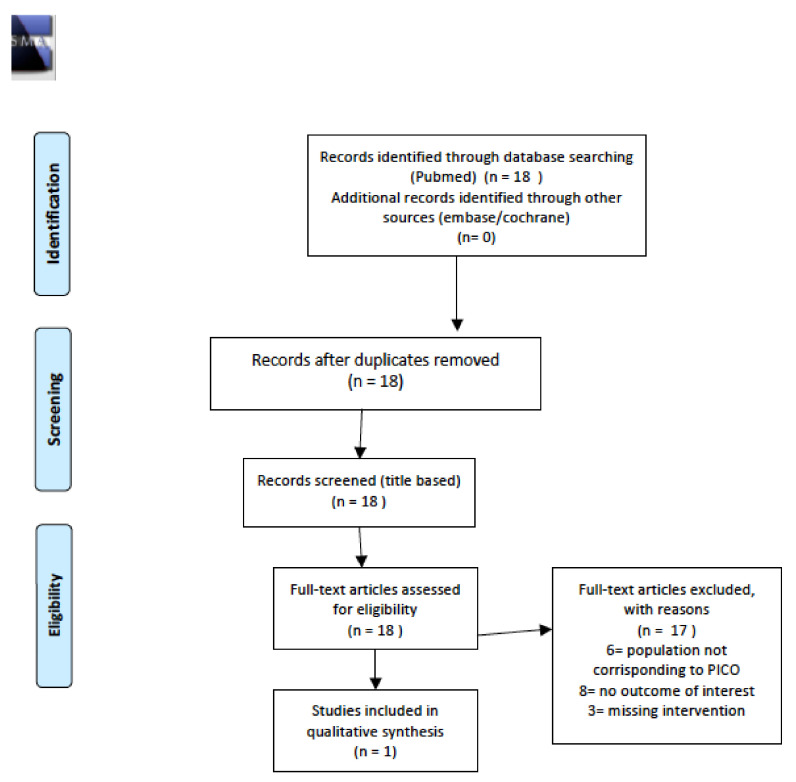
Prisma flow-chart metabolic syndrome incidence.

The initial search revealed a total of 18 abstracts, of which 17 were excluded after full-text evaluation as they were not pertinent to the study objectives. Thus, only one study was ultimately included in this review. Details of the whole screening process, including reasons for full-text exclusion, are reported in Figure 10.

The study by Daniele et al. [16] aimed to evaluate the metabolic risk in NHL survivors by focusing on unhealthy lifestyles (i.e., poor nutrition, smoking, physical inactivity), weight gain and changes in body composition and clinical features such as type of treatment and high-dose steroid use. This prospective cohort study was conducted from November 2016 to September 2020; enrolled were 60 consecutive NHL survivors (34 women and 26 men) in remission for at least three years and in follow-up. The most representative histotype was DLBCL (95%); only 5% of the cases were advanced-stage follicular lymphoma. All enrolled patients (100%) had been treated with CHOP-like regimens which included high-dose steroids. The authors of this study reported MetSyn in 60% of patients and a higher waist circumference value in patients with MetSyn versus those without, both in females and in males mean value (98 ± 17 vs. 84 ± 11, *p* = 0.001 and mean value 104 ± 9.0 vs. 93 ± 8.1, *p* = 0.005, respectively). Two other significant findings were: (i) the change in body composition among survivors with MetSyn in comparison with those without MetSyn. In particular, a significant decrease in sarcopenia was observed (67.9% vs. 71.7%, respectively; *p* = 0.002); and (ii) an increased MetSyn risk associated with steroid use administered with first- and second-line chemotherapy (hazard ratio (HR) = 6.80; *p* = 0.096). Concerning the correlation with unhealthy lifestyles, unbalanced diet and low physical activity appeared to be associated with a higher risk of developing metabolic syndrome (HR = 0.30, *p* = 0.02 and HR = 0.32, *p* = 0.01, respectively). The authors suggested the fundamental importance of including monitoring nutritional status and a correct lifestyle of long-term lymphoma survivors to prevent the onset of MetSyn and correlated diseases.

According to the NOS, the quality of the manuscript was adequate on all points of the checklist [16].

#### 3.4.2. Efficacy of Planned Follow-Up Schemes to Diagnose Metabolic Syndrome and Related Sarcopenia in Long-Term cHL or DLBCL Survivors Treated with First- and/or Second-Line CT/RT and ASCT?

The research strategy produced no abstract corresponding to the PICOs on the early detection or follow-up of MetSyn in this subset of patients.

## 4. Discussion

With the present systematic review, FIL researchers wanted to collect the evidence available on the main long-term endocrine-metabolic sequelae of patients treated for cHL or DLBCL, with the aim of both estimating the problem size and of indicating possible models of planned follow-up for early diagnosis of asymptomatic patients. (Table 5) The considered sequelae were functional and morphological alterations of the thyroid gland, functional gonadal and bone metabolism alterations and MetSyn and associated sarcopenia. This research was driven by the current lack of structured, evidence-based recommendations for the subset of long-term cHL and DLBCL survivors treated in adulthood.

The systematic review of the literature regarding the alterations in thyroid function and morphology revealed studies including only long-term cHL survivors. The majority of these studies was conducted with chemotherapic regimens that are no longer used in daily practice; however, late toxicities of these treatments are still observed in long-term follow-up of lymphoma survivors. Hypothyroidism, either subclinical or overt, is the main alteration found in this patient population. The prevalence of hypothyroidism in cHL survivors treated in adulthood is higher than that of the general population and has been reported in up to 60% of patients after first-line treatment, depending on the definition used and the population studied [26,27,28]. However, most studies report an average prevalence of 30%, and that it generally occurs within 10 years from RT treatment on the neck and surrounding structures.

Neck RT is the main risk factor for hypothyroidism and may play a role in the development of late thyroid dysfunction or thyroid cancer; the role of chemotherapy, instead, has yet to be clarified. In fact, some correlations between thyroid dysfunction and radiation dose have been recorded in several series of adult survivors of childhood cHL, where the prevalence of hypothyroidism was linearly correlated to the delivered radiation dose to the thyroid gland. A prevalence of hypothyroidism of 30% was associated with a radiation dose of 35–49 Gy and up to 50% with a radiation dose of >50 Gy [33]. Moreover, the relative risk for thyroid diseases was estimated to increase by 1.02 for each delivered Gy to the thyroid gland [34]. The current multimodal treatment for cHL and DLBCL is based on the use of modern radiotherapy characterized by a progressive reduction in radiation dose and volume, from involved-field RT (IFRT) to involved-nodal/involved site RT (INRT/ISRT) [20,21]. Reducing the dose and the volume of thyroid radiation may therefore reduce the prevalence of thyroid dysfunctions in patients undergoing IFRT. The introduction of intensity-modulated radiation therapy (IMRT) and ISRT was also investigated to assess the prevalence of thyroid dysfunctions. However, a recent study reported hypothyroidism in 40–66% of cHL patients treated with the reduced-field (ISRT) and IMRT technique. In particular, the incidence of hypothyroidism was higher if dosimetric constraints (for example, if thyroid V30 was less than 62% or if 2.2 mL of thyroid gland received less than from 25 Gy) were not achieved, even if IMRT was employed [29]. Thus, even with modern radiotherapy approaches, the incidence of thyroid dysfunction, mainly hypothyroidism, seems not to be negligible in long-term lymphoma survivors treated with radiation therapy on the neck/mediastinum.

Hyperthyroidism is much less frequent in long-term survivors of cHL, with only Grave’s disease reported in a few patients.

Of note, we found no incidence/prevalence data for thyroid dysfunction in DLBCL patients treated with first-line treatment nor in either cHL or DLBCL patients undergoing ASCT. However, findings from pediatric and mixed series suggest that this could be an issue for patients treated in adulthood as well [35].

As a result, the need for long-term follow-up of thyroid dysfunction in lymphoma survivors is increasingly recognized and addressed through the publication of surveillance guidelines. Indeed, the National Comprehensive Cancer Network (NCCN) and the Dutch “Better care after Hodgkin lymphoma: Evaluation of long-term Treatment Effects and screening Recommendations” (BETER) consortium specifically provides guidelines for monitoring late effects in cHL survivors, including an annual TSH measurement in patients with a history of neck irradiation [11,36].

Secondary thyroid cancer is another adverse event historically correlated with neck irradiation, with a cumulative incidence of 0.5% by age 45, representing an 18-fold increase in risk compared to the general population. In particular, female patients and patients irradiated when younger than 20 years are considered at higher risk, while no case of thyroid malignancy has been recorded in a patient over age 35 at the time of neck irradiation [37,38,39,40]. However, no article dealing with thyroid nodules or thyroid cancer fully complied with our predetermined search criteria.

In a cohort of 585 survivors of childhood, adolescent and young adult cancer (cHL 34%, NHL 7.5%) who were exposed to neck RT and followed for a median of 3.1 years, 40 (6.8%) had an abnormal thyroid physical exam, 36 (6.2%) had a thyroid nodule detected on ultrasound, 24 (4.1%) were referred to fine needle aspiration (FNA) after sonographic evaluation, and nine (1.5%) ultimately underwent either total or partial thyroidectomy, which revealed papillary thyroid carcinoma in seven patients (1.2%) [40]. Accordingly, the authors recommend annual palpation of the thyroid as a screening method for thyroid cancer in survivors with a history of RT to the neck or surrounding structures, with further investigations to be done in high-risk individuals with abnormalities on thyroid physical exam. The American Thyroid Association guidelines on thyroid nodules and cancer also state that there is insufficient evidence to endorse ultrasound screening in cancer survivors previously exposed to neck RT [41]. In contrast with this position, some professional society guidelines, such as those issued jointly by the American Association of Clinical Endocrinologists, the Italian Associazione Medici Endocrinologi and the European Thyroid Association [42,43], recommend performing ultrasound evaluation in selected patients who are at risk of thyroid malignancy, including patients with a prior history of head and neck irradiation.

Based on the evaluated papers, FIL researchers agree that cHL survivors who have received RT on the neck and surrounding structures should be regarded as a population at risk of hypothyroidism therefore requiring annual measurements of serum TSH, FT3 and FT4 levels for early identification of thyroid dysfunction. Prolonged elevation of TSH levels correlates with significant long-term morbidity, including increased total low-density lipoprotein cholesterol levels and atherosclerotic events [44]. Accordingly, international guidelines recommend thyroid hormone replacement therapy to be considered in adults whose TSH levels are above 10 mIU/L, as well as when TSH levels are lower if the patient is young, symptomatic or has other indications for prescribing this therapy, such as cardiovascular disease or antibodies to thyroid peroxidase [45].

Despite the lack of evidence specifically supporting the superiority of ultrasound versus the palpatory clinical examination of the thyroid gland to screen for thyroid nodules in lymphoma survivor populations, as most societies of endocrinology endorse ultrasound evaluation in patients with a prior history of neck irradiation, and given the favorable cost, widespread availability and the extraordinary “patient-friendliness” of ultrasonography, FIL researchers agree that thyroid ultrasound imaging should be performed in all lymphoma patients being exposed to neck RT.

The indication for FNA should take into account the patient’s risk factors for malignancy and the sonographic and structural risk characteristics of the nodule and the clinical judgment of the treating team as recommended by international endocrinology guidelines.

Gonadal toxicity following cancer therapy mainly involves a decline in the endocrine functions that sustain the production of sex hormones (testosterone and estrogens) and germ cells (ova and sperm) [46]. As the impact of gonadal toxicity on female and male fertility is the subject of a systematic review in the present series [15], this review focused on the treatment-related decline in gonadal sex hormones and on the impact of new chemo- and/or radiotherapy modalities.

The testis and ovaries are the most radiosensitive tissues. Permanent ovarian impairment can be detected after 5–6 Gy, with higher radiosensitivity related to older patients [47]. Permanent azoospermia was seen after 4 Gy, while the testosterone-producing Leydig cells were less radiosensitive, with a dose constraint of 24 Gy in prepuberty and over 24 Gy in postpuberty [48]. Gonadal dysfunction following radiotherapy may occur when the gonads are close to or within the radiation field. In the 1980s and 1990s, EFRT in association with agents such as mechlorethamine, cyclophosphamide, or procarbazine were held responsible for the gonadal damage in patients with cHL [49]. Historically, in fact, the inverted-Y radiation technique that included the pelvis was used alone or as part of total nodal irradiation in outdated cHL treatments. After the introduction of combined treatment (chemotherapy and EFRT), several studies underlined the concept that, in addition to infradiaphragmatic radiotherapy, the use of alkylating agents such as procarbazine may have explained the gonadal toxicities observed in cHL and NHL patients [50,51,52,53]. In a series of young male and female cHL patients treated with MVPP between 1980 and 1983, high incidences of azoospermia (all male patients up to 10 years after treatment) and menopause (50%) were recorded [30]. Moreover, in the study by Meissner et al., after a median follow-up of 14 years, menopause occurred significantly earlier in 46 women with aggressive NHL compared to the general population (47 versus 51 years, *p* < 0.0001) [31]. Thus, although fertility did not show a dramatic decline, late impairment of ovarian function was recorded in a considerable proportion of the women. With the introduction of combined chemotherapy and IFRT in the last few decades, pelvic irradiation is rarely administered for cHL and DLBCL. Given this, no retrospective or prospective study has been published in recent years that compared EFRT and IFRT or IFRT and INRT/ISRT in terms of the prevalence of gonadal toxicity. If pelvic radiotherapy is performed, oophoropexy (surgical removal of the ovaries to the midline behind the uterus or high up at the pelvic brim, away from the field of radiation therapy) and/or ovarian shielding can be proposed to very selected young female patients [54]. Male patients, instead, can have testicular shielding, which can reduce the dose below that which causes gonadal damage

Based on the results of the systematic review, FIL researchers suggest that the patient’s age, reproductive intentions and symptoms must always be taken into account to guide exams. In female patients of childbearing age and with reproductive intentions, fertility status should be assessed with hormone tests and ultrasound (please see the dedicated systematic review on fertility preservation and follow-up for this patient population) [15]. Hormone blood tests with LH, FSH and estradiol measurement may also be recommended after a clinical evaluation of symptomatic patients with hot flashes, amenorrhea and/or irregular periods [55,56].

As indicated above, men undergoing chemotherapy or gonadal irradiation are at an increased risk of testicular failure. Low testosterone levels may lead not only to sexual dysfunction but also to an increased risk of osteoporosis and metabolic and cardiovascular diseases. Accordingly, LH, FSH, total testosterone and SHBG blood tests should be offered to male lymphoma survivors as a part of their regular follow-up to detect any possible signs of androgen deficiency. For this population, see the systematic review on fertility in this same series [15].

In adult lymphoma patients, systemic chemotherapy has been frequently associated with skeletal damage due to the cytotoxic effect of chemotherapeutic agents on osteoblasts, which results in the reduction in bone volume and new bone formation and which can be exacerbated by glucocorticoid treatment [32,57,58]. Of note, cyclophosphamides may increase bone resorption primarily through gonadal damage, while the direct inhibition of bone formation has been reported with doxorubicin [59,60]. As part of most therapeutic regimens for lymphomas, glucocorticoid agents may also exert adverse effects on the trabecular and cortical bone microstructure, with the loss in bone mass being greater in the spine and proximal femur than in the forearm and the femoral shaft [60]. Due to decreased bone mass and changes in bone microarchitecture, therefore, higher fracture rates are expected in lymphoma patients undergoing chemotherapy regimens including glucocorticoids.

The only study included in this systematic review to assess the incidence of bone mineral damage focused on DLBCL survivors treated with R-CHOP or CHOP-like chemotherapy. Svendsen et al. reported reduced vertebral bone density over time and a fracture risk of 14% over two years, which exceeds the annual risk of 2% reported for the population age ≥ 50 years in Denmark [32]. No article fully matched our search criteria for cHL. However, in the Bone Marrow Transplant Survivor Study, 92 cHL patients who had undergone autologous hematopoietic stem cell transplant (AHSCT) at a median age of 32 years (range 13–54) reported significantly higher frequencies of osteoporosis than did their sibling controls after a median follow-up of six years [35].

Although it is well recognized that ionizing radiation (IR) can induce bone loss, which consequently increases the risk of fractures, no data on lymphoma patients have been published to this point. In a retrospective series of non-hematological cancer patients, the following dose–response relationship between radiation delivered to the vertebral body and a bone mineral density loss in the field of radiation was found: 5 Gy, 15 Gy, 25 Gy, 35 Gy and 45 Gy caused a 21.7%, 31.1%, 40.5%, 49.9% and 59.3% decrease, respectively, in bone attenuation (in Hounsfield units) following RT [61]. In the few last decades, with the modern radiotherapy approach (reduction in radiation volume and total radiation dose) spine, limb or hip volume included in the radiation field may be reduced as compared to the more outdated treatment. It may be useful for ongoing and future studies to record the dose to the bone and to try to reduce bone irradiation as much as possible.

Despite the higher risk of bone disease, there are no dedicated screening schedules in long-term lymphoma survivors. The United States Preventive Services Taskforce recommends screening for osteoporosis with dual-energy X-ray absorptiometry (DXA) of the hips and lumbar spine to prevent osteoporotic fractures in all women 65 years or older and in women younger than 65 years at increased risk of osteoporosis, as determined by a clinical risk assessment tool; evidence is still lacking for men [62]. As lymphoma is often diagnosed in individuals age 60 or over, the age-related decline of gonadal hormones may contribute to bone loss. However, treatment-related hypogonadism and vitamin D deficiency may cause sustained bone mineral loss even in younger individuals [63]. Accordingly, surveillance for premature ovarian failure and hypogonadism may be appropriate to identify lymphoma patients at higher risk of bone demineralization or osteoporosis.

As a result of this systematic review, the FIL researchers have identified two populations at risk of developing severe osteoporosis and fractures: (i) patients aged > 60 years who have received high-dose steroids, including treatment such as R-CHOP or second-line treatments, for example, R-DHAP (dexamethasone-high-dose cytarabine-cisplatin), IGEV (ifosfamide-gemcitabine-vinorelbine-prednisone) and ASCT, and radiotherapy, and (ii) hypogonadal patients of all ages. While DXA is the standard method for diagnosing alterations of bone metabolism, computed tomography can also provide direct information. It is advisable to diagnose bone alterations in early follow-up to begin remineralization therapy to prevent fractures, but the optimal timing for the execution of DXA is not defined. Recently, introducing vitamin D during R-CHOP chemotherapy has been advised in deficient patients, but its long-term efficacy in preventing bone damage and fractures is currently unknown.

MetSyn is associated with an increased risk of type 2 diabetes mellitus (T2DM) and cardiovascular disease. The most widely used definition of metabolic syndrome is that of the National Cholesterol Education Program-Adult Treatment Panel III [64,65]. The five screening variables used to identify this disease are waist circumference, higher circulating levels of triglycerides and low-density lipoprotein cholesterol, fasting glycaemia and blood pressure. Metabolic disorders represent an emerging long-term sequelae of anticancer treatment in cancer survivors. Chemotherapy appears to contribute to the pathophysiology of cancer treatment–induced metabolic syndrome, mainly through disturbances of the hypothalamic-pituitary axis and their target organs; deficiencies of the growth and gonadal hormones represent the early stages of the development of this syndrome [66,67,68,69]. Some historical series have reported a higher risk of T2DM after abdominal irradiation for both cHL and NHL [70,71,72]. Currently, in contemporary multimodal treatments of lymphoma patients, para-aortic or abdominal irradiation is infrequent. In long-term lymphoma survivors, cancer treatments are associated with metabolic risk and weight gain. Changes in body composition and lean mass loss are often called sarcopenic obesity, a clinical-functional condition characterized by an alteration of the composition of the body where there is an excess of fat mass and a lack of muscle mass (sarcopenia). In sarcopenic obesity, the pathophysiology of sarcopenia and that of obesity are strongly interconnected; the evidence suggests that fat-free mass (FFM) and fat mass are subject to the so-called one quarter rule: for any increase in body fat, a parallel variation of FFM occurs, corresponding approximately to 25% [73]. The loss of mass and muscle function can also be favored by obesity as an independent risk factor due to related alterations: chronic low-grade inflammation, an increase in oxidative stress, insulin resistance (with a decrease in the anabolic capacity of the musculature striata, in addition to the worsening of the carbohydrate profile), a sedentary lifestyle and the higher incidence of chronic diseases, which can have a negative impact on catabolism muscle [74]. Chronic low-grade inflammation, present in obesity and favored by it, causes decreased muscle anabolic function, which is mediated by variations in the production of factors such as TNF, IL-6, leptin and GH [75]. The onset of MetSyn and sarcopenia in these patients have unfortunately not yet been investigated, and currently, there are very few studies on this topic; and there are no adequate nutrition guidelines for lymphoma survivors. In this review, only one paper was included, and from it it appears that metabolic disorders and a progressive loss of lean mass are common in DLBCL survivors, while data on cHL are still lacking.

According to the available evidence, FIL researchers have agreed to consider at risk of MetSyn, sarcopenic obesity and related disorders those DLBCL survivors treated with chemotherapy including high-dose steroids. For these patients, nutritional monitoring and an unhealthy lifestyle correction should be included in the follow-up [76,77]. The correct timing for nutritional assessment and monitoring has not yet been established, but the authors encourage at least one nutritional assessment during follow-up, including bioelectrical vettorial impedance analysis (BIVA) to detect early states of malnutrition, sarcopenia and morbid obesity. Providing personalized food plans and implementing targeted lifestyle and eating habit interventions should ensure that these cancer survivors receive a balanced supply of macronutrients (carbohydrates, proteins and lipids) and micronutrients (vitamins and minerals), especially in cases of inappropriate diet, which can cause rapid, repeated weight gain with an inadequate recovery of lean mass.

## 5. Conclusions

Given the progressive improvement in the overall survival of cHL and DLBCL patients, several treatment-related long-term toxicities, including endocrine and metabolic sequelae, have come to the attention of multidisciplinary team of health care professionals (Table 6).

In the present manuscript, the authors highlighted the evidence emerging from a detailed systematic review of the literature tailored on cHL and DLBCL, the two main histotypes of lymphoma survivors. The authors have circumscribed the field of search to the principal chemotherapic strategies: ABVD, R-CHOP, ASCT, RT, in order to propose homogeneous and actualized conclusions.

Dedicated screening programs for the early diagnosis of thyroid disease, gonadal dysfunction, osteoporosis and metabolic syndrome are recommended in high-risk patients, although there are currently no validated strategies.

## Figures and Tables

**Table 1:** Clinical questions and PICOs addressed by the review: Late thyroid sequalae and long-term monitoring.

Clinical Question	PICOs
What is the incidence or prevalence of thyroid diseases in long-term cHL or DLBCL survivors treated with first- and/or second-line CT/RT and ASCT?	P: population of long-term cHL or DLBCL survivors (≥5 years disease-/treatment-free) aged ≥ 18 years at diagnosis I: chemotherapy (e.g., ABVD for cHL; RCHOP for DLBCL), radiotherapy (neck and/or mediastinum) C1: none C2: general population matched for age and sex C3: other CT/RT regimens O1: incidence or prevalence of abnormal thyroid function tests (overt or subclinical hypothyroidism, hyperthyroidism) O2: autoimmune thyroid disease (Hashimoto’s thyroiditis, Graves’ disease) O3: thyroid nodules (single nodule, multiple nodules, thyroid cancer)
Treatment comparisons	
Has the incidence or prevalence of thyroid diseases in in long-term cHL or DLBCL survivors treated with first- and/or second-line CT/RT and ASCT changed with the introduction of modern radiotherapy?	P: population of long-term cHL or DLBCL survivors (≥5 years disease-/treatment-free) aged ≥ 18 years at diagnosis I: new RT approaches (3DCRT, IMRT, dose/volume reduction) C: previous RT regimens (2DRT, Extended Field RT) O: number of cases of abnormal thyroid function test, autoimmune thyroid disease, nodular pathology of the thyroid, thyroid cancer
Optimal follow-up	
Efficacy of planned follow-up schemes to early diagnose thyroid diseases in long-term cHL or DLBCL survivors treated with first and/or second line CT/RT and ASCT	P: population of long-term cHL or DLBCL survivors (≥5 years disease-/treatment-free) aged ≥ 18 years, particularly patients at risk (e.g., neck and/or mediastinum RT) I: scheduled follow-up/monitoring planned (e.g., annual thyroid function test, antithyroid autoantibody assay, ultrasound, ultrasound-guided fine needle aspiration, etc.) C1: no follow-up/monitoring planned C2: follow-up/monitoring planned with different intensity (with respect to I, including different components, timing and frequencies) O: incidence or prevalence of abnormal thyroid function test, autoimmune thyroid disease, nodular thyroid disease, thyroid cancer, problems related to overdiagnosis, quality of life, mortality

cHL, classical Hodgkin lymphoma; DLBCL, diffuse large B-cell lymphoma; CT, chemotherapy; RT, radiotherapy; 2DRT, two-dimensional radiation therapy; 3DCRT, three-dimensional conformal radiation therapy; IMRT, intensity-modulated radiation therapy; ASCT, autologous stem cell transplant; ABVD, Adriamycin (doxorubicin)-bleomycin-vinblastine-dacarbazine; R-CHOP:rituximab-doxorubicin-cyclophosphamide-vincristine-prednisone; P, population; I, intervention; C, control; O, outcome.

**Table 2:** Clinical questions and PICOs addressed by the review: Late gonadal sequalae and long-term monitoring.

Clinical Question	PICOs
What is the incidence or prevalence of late gonadal sequalae in long-term cHL or DLBCL survivors treated with first- and/or second-line CT/RT and ASCT?	(a). P: population of long-term cHL or DLBCL survivors (≥5 years disease-/treatment-free) aged ≥ 18 years at diagnosis I: chemotherapy (e.g., ABVD for HL; RCHOP for DLBCL), pelvic radiotherapy C1: none C2: general population matched for age and sex C3: other CT/RT treatment regimens O: incidence or prevalence of abnormal testosterone secretion (low serum testosterone and/or elevated LH levels) (b). P: population of long-term cHL or DLBCL survivors (≥5 years disease-/treatment-free) aged ≥ 18 years at diagnosis I: chemotherapy (e.g., A BVD for cHL; RCHOP for DLBCL), pelvic radiotherapy C1: none C2: general population matched for age and sex C3: other CT/RT treatment regimens O: incidence or prevalence of symptoms of estrogen deprivation (e.g.,hot flashes, vaginal dryness, dyspareunia)
Treatment comparisons	
Has the incidence or prevalence of late gonadal sequalae in long-term cHL or DLBCL survivors treated with first- and/or second-line CT/RT and ASCT changed with the introduction of modern radiotherapy?	(a). P: population of long-term cHL or DLBCL survivors (≥5 years disease-/treatment-free) aged ≥ 18 years at diagnosis I1: new radiotherapy approaches (3DCRT, IMRT, dose/volume reduction,) C: previous radiotherapy regimens 2DRT, Extended Field RT) O: number of cases of abnormal testosterone secretion (low serum testosterone and/or elevated LH levels) (b). P: population of long-term cHL or DLBCL survivors (≥5 years disease-/treatment-free) aged ≥ 18 years at diagnosis I1: new radiotherapy approaches (3DCRT, IMRT, dose/volume reduction,) C: previous radiotherapy regimens (2DRT, Extended Field RT) O: number of cases of secondary (transient, permanent) amenorrhea, number of patients reporting symptoms of estrogen deprivation
Optimal follow up	
Efficacy of planned follow-up schemes in the diagnosis of late gonadal dysfunction in long-term cHL or DLBCL survivors treated with first and/or second line CT/RT and ASCT	(a) P: population of long-term cHL or DLBCL survivors (≥5 years disease-/treatment-free) aged ≥ 18 years at diagnosis, particularly patients at risk (e.g., pelvic RT) I: scheduled follow-up/monitoring (e.g., annual total testosterone dosage, LH, annual spermiogram run, etc.) C1: no follow-up/monitoring planned C2: follow-up/monitoring planned with different intensity (with respect to I, including different components, times and frequencies) O: alterations in testosterone secretion, problems related to overdiagnosis, quality of life, mortality (b) P: population of long-term cHL or DLBCL survivors (≥5 years disease-/treatment-free) aged ≥ 18 years at diagnosis, particularly patients at risk (e.g., pelvic RT) I: scheduled follow-up/monitoring (e.g., annual hormone test dosage—FSH, LH, estradiol, inhibin B, AMH, progesterone—and annual transvaginal pelvic ultrasound) C1: no follow-up/monitoring planned C2: follow-up/monitoring planned with different intensity (with respect to I, including different components, times and frequencies) O: secondary amenorrhea, problems related to overdiagnosis, quality of life, mortality

cHL, classical Hodgkin lymphoma; DLBCL, diffuse large B-cell lymphoma; CT, chemotherapy; RT, radiotheraoy; 2DRT, two-dimensional radiation therapy; 3DCRT, three-dimensional conformal radiation therapy; IMRT, intensity-modulated radiation therapy; ASCT, autologous stem cell transplant; ABVD, Adriamycin (doxorubicin)-bleomycin-vinblastine-dacarbazine;R-CHOP:rituximab-doxorubicin-cyclophosphamide-vincristine-prednisone; LH luteinizing hormone; FSH: follicle-stimulating hormone; AMH: anti-Müllerian hormone; P, population; I, intervention; C, control; O, outcome.

**Table 3:** Clinical questions and PICOs addressed by the review: Late bone disease and long-term monitoring.

Clinical Question	PICOs
What is the incidence or prevalence of changes in bone quality and bone mineral density in long-term cHL or DLBCL survivors treated with first and/or second line CT/RT and ASCT	P: population of long-term cHL or DLBCL survivors (≥5 years disease-/treatment-free) aged ≥ 18 years at diagnosis I: CT (e.g., ABVD for cHL; R-CHOP for DLBCL), RT C1: none C2: general population matched for age and sex C3: other CT/RT treatment regimens O: prevalence and/or incidence of osteopenia, osteoporosis, fractures
Treatment comparisons	
(3) Has the incidence or prevalence of changes in bone quality and mineral density in long-term cHL or DLBCL survivors treated with first- and/or second-line CT/RT and ASCT changed with the introduction of modern radiotherapy?	P: population of long-term cHL or DLBCL survivors (≥5 years disease-/treatment-free) aged ≥ 18 years at diagnosis I: new RT approaches (3DCRT, IMRT, dose/volume reduction,) C: previous RT regimens (2DRT, extended field RT) O: number of cases of osteopenia, osteoporosis, fractures
Optimal follow up	
Efficacy of planned follow-up schemes in diagnosing changes in bone mineral density and quality in long-term cHL or DLBCL survivors treated with first and second line CT/RT and ASCT	P: population of long-term cHL or DLBCL survivors (≥5 years disease-/treatment-free) aged ≥ 18 years at diagnosis, particularly patients at risk (e.g., RT) I: Scheduled follow-up/monitoring planned (e.g., annual calcium and vitamin D dosage, annual bone densitometry) C1: no follow-up/monitoring planned C2: follow-up/monitoring planned with different intensity (with respect to I, including different components, timing and frequencies) O: osteoporosis, osteopenia, fracture, quality of life, mortality

cHL, classical Hodgkin lymphoma; DLBCL, diffuse large B-cell lymphoma; CT, chemotherapy; RT, radiotherapy; 2DRT, two-dimensional radiation therapy; 3DCRT, three-dimensional conformal radiation therapy; IMRT, intensity-modulated radiation therapy; ASCT, autologous stem cell transplant; ABVD, Adriamycin (doxorubicin)-bleomycin-vinblastine-dacarbazine; R-CHOP, rituximab-doxorubicin-cyclophosphamide-vincristine-prednisone; P, population; I, intervention; C, control; O, outcome.

**Table 4:** Clinical questions and PICOs addressed by the review: incidence of metabolic syndrome.

Clinical Question	PICOs
What is the incidence of metabolic syndrome in long-term cHL or DLBCL survivors treated with first- and/or second-line CT/RT and ASCT?	P: population of long-term cHL or DLBCL survivors (≥5 years disease-/treatment-free) aged ≥ 18 years at diagnosis I: chemotherapy (e.g., ABVD for cHL; RCHOP for DLBCL), radiotherapy C1: none C2: general population matched for age and sex C3: other CT/RT treatment regimens O: incidence of metabolic syndrome
Optimal follow up	
What is the efficacy of planned follow-up schemes to diagnose metabolic syndrome and related sarcopenia in in long-term cHL or DLBCL survivors treated with first- or second-line CT/RT and ASCT?	P: population of long-term (≥5 years disease- or/treatment-free) cHL or NHL (DLBCL in particular) aged ≥ 18 years at diagnosis I: chemotherapy (e.g., ABVD for cHL; RCHOP for NHL/DLBCL), radiotherapy C1: general population matched for age and sex C2: autologous stem cell transplant (ASCT), high dose steroids O1: follow-up of metabolic syndrome or metabolic risk O2: long-term nutritional effects such as change of body composition, visceral obesity and weight, sarcopenia and/or dysplipidemia

cHL, classical Hodgkin lymphoma; DLBCL, diffuse large B-cell lymphoma; CT, chemotherapy; RT, radiotherapy; ASCT, autologous stem cell transplant; P, population; I, intervention; C, control; O, outcome.

**Table 5:** Summary of findings.

Thyroid Diseases			
PICO A: Thyroid Diseases Incidence and Prevalence			
Study	Study Design and Sample Size	Intervention & Comparison	Outcomes
Enrici RM, 1999 [26]	Prospective RCT (73 pts)	RT/CT 36 pts RT 37 pts	Prevalence of hypothyroidism 2/73 = 2.7%, hypothyroidism in the CT/RT subgroup (2/36 = 5.5%)
Illes, 2003 [28]	Retrospective cohort study (151 cHL patients)	RT, CT or both	Prevalence of hypothyroidism, 26.5% prevalence of hyperthyroidism 0.01%, prevalence of antibodies 18%
Bethge W, 2000 [27]	Retrospective cohort study (177 cHL patients)	RT, CT or both	prevalence of hypothyroidism, 27% prevalence of subclinical hypothyroidism, 20% prevalence of overt hypothyroidism 7%
**PICO B: treatment comparison**			
Pinnix, 2018 [29]	Retrospective cohort study (140 cHL patients)	RT IMRT (ISRT, mediastinum with-without neck) (90 pts) RT 3DCRT (ISRT, mediastinum with-without neck) (50 pts)	three-year rates of freedom from hypothyroidism of 56.1% for the 3D-CRT group and 40% for the IMRT group (*p* = 0.057)
**GONADAL DYSFUNCTIONS**			
**PICO A: Gonadal dysfunctions incidence and prevalence**			
**Study**	**Study design and sample size**	**Intervention & Comparison**	**Primary outcomes**
King, 1985 [30]	Retrospective (N° not reported cHL)	CT ± RT	prevalence of azoospermia 100%
Meissner, 2015 [31]	Retrospective (46 NHL pts)	CT	no/few menopausal symptoms 25.7% mild menopausal symptoms 17.1% moderate menopausal symptoms 37.1% severe menopausal symptoms 20%
**BONE DISEASES**			
**PICO A: Bone diseases incidence and prevalence**			
**Study**	**Study design and sample size**	**Intervention & Comparison**	**Primary outcomes**
Svendsen, 2017 [32]	Retrospective (111, NHL pts)	R-CHOP (-like)	prevalence of fractures 14%
**METABOLIC SYNDROME**			
**PICO A: Metabolic Syndrome incidence and prevalence**			
**Study**	**Study design and sample size**	**Intervention & Comparison**	**Primary outcomes**
Daniele, 2021 [16]	Prospective cohort (60 NHL pts)	CT	presence of metabolic syndrome in 60% of patients

CT, chemotherapy; RT, radiotherapy; cHL, classic Hodgkin Lymphoma; NHL Non-Hodgkin Lymphoma

**Table 6:** Highlights emerged from the systematic review and expert panel advices: planned follow-up schemes to early diagnose thyroid, gonadal, bone dysfunction and metabolic syndrome in cHL and DLBCL survivors.

Risk Category	Suggested Follow-Up
All patients receiving RT on the neck and surrounding structures are at high risk of hypothyroidism	Thyroid evaluation with: - annual measurement of TSH, FT3, FT4 - Thyroid ultrasound FNA should be performed according to international endocrinology guidelines
Patients with reproductive intentions and symptoms related to gonadal dysfunction due to CT or RT	Female patients: measurement of LH, FSH and estradiol; ultrasound Male patients: measurement of LH, FSH, total testosterone and SHBG
Patients aged > 60 years treated with high-dose steroids, second-line treatments, ASCT and hypogonadal patients of all ages are at high risk of osteoporosis and bone fractures	DXA ± tomography scan to detect bone alterations are recommended in early follow-up, although optimal timing is unknown
DLBCL survivors treated with chemotherapy including high-dose steroids are at increased risk of metabolic syndrome and associated sarcopenic obesity	Nutritional assessment and monitoring at least once during follow-up (BIVA) Personalized food plans Unhealthy lifestyle correction

RT, radiotherapy; CT, chemotherapy; DLBCL, diffuse large B cell Lymphoma; ASCT, autologous stem cell transplant; FNA, fine needle aspiration; BIVA, bioelectrical impedance vector analysis.

## Supplementary Materials

The following supporting information can be downloaded at: https://www.mdpi.com/article/10.3390/cancers14061439/s1. Supplementary Material File S1: Example of search strategy used in MedLine and adapted to search the other databases; Supplementary Material File S2: Full text eligibility and data extraction; Supplementary Material File S3: Risk of bias.

## References

[1] National Comprehensive Cancer Network Guidelines version 2.2020 “Survivorship”. https://www.nccn.org/professionals/physician_gls/pdf/survivorship.pdf.

[2] Haematological Malignancy Research Network (HMRN). https://www.hmrn.org/statistics/survival.

[3] National Comprehensive Cancer Networ Guidelines version 4.2021 “Hodgkin Lymphoma”. https://www.nccn.org/professionals/physician_gls/pdf/hodgkins.pdf.

[4] National Cancer Institute: Surveillance, Epidemiology, and End Results Program. https://seer.cancer.gov/statfacts/html/dlbcl.html.

[5] Tilly H., Gomes da Silva M., Vitolo U., Jack A., Meignan M., Lopez-Guillermo A., Walewski J., André M., Johnson P.W., Pfreundschuh M. (2015). ESMO Guidelines Committee. Diffuse large B-Cell Lymphoma (DLBCL): ESMO Clinical Practice Guidelines for diagnosis, treatment and follow-up. Ann. Oncol..

[6] National Comprehensive Cancer Networ Guidelines version 4.2021 “B-cell lymphomas”. https://www.nccn.org/professionals/physician_gls/pdf/b-cell.pdf.

[7] AIRTUM 2020: I Numeri del Cancro in Italia. www.registri-tumori.it.

[8] Liu Y., Barta S.K. (2019). Diffuse large B cell lymphoma: 2019 update on diagnosis, risk stratification and treatment. Am. J. Hematol..

[9] Minoia C., Bari A., Nassi L., Banzi R., Gerardi C., Lenti V., Calabrese M., Spina M., Guarini A. (2021). Management of lymphoma survivor patients in Italy: An evaluation by Fondazione Italiana Linfomi. Tumori J..

[10] Ciavarella S., Minoia C., Quinto A.M., Oliva S., Carbonara S., Cormio C., Cox M.C., Bravo E., Santoro F., Napolitano M. (2017). Improving Provision of Care for Long-term Survivors of Lymphoma. Clin. Lymphoma Myeloma Leuk..

[11] Ng A.K., van Leeuwen F.E. (2016). Hodgkin Lymphoma: Late Effects of Treatment and Guidelines for Surveillance. Semin. Hematol..

[12] Gerardi C., Allocati E., Minoia C., Guarini A., Banzi R. (2021). Long-Term Follow-Up of Classical Hodgkin Lymphoma and Diffuse Large B-Cell Lymphoma Survivors: Aims and Methodological Approach for Fondazione Italiana Linfomi Systematic Reviews. Cancers.

[13] Minoia C., Gerardi C., Allocati E., Daniele A., De Sanctis V., Bari A., Guarini A. (2021). The Impact of Healthy Lifestyles on Late Sequelae in Classical Hodgkin Lymphoma and Diffuse Large B-Cell Lymphoma Survivors. A Systematic Review by the Fondazione Italiana Linfomi. Cancers.

[14] Franceschetti S., Annunziata M.A., Agostinelli G., Gerardi C., Allocati E., Minoia C., Guarini A. (2021). Late Neurological and Cognitive Sequelae and Long-Term Monitoring of Classical Hodgkin Lymphoma and Diffuse Large B-Cell Lymphoma Survivors: A Systematic Review by the Fondazione Italiana Linfomi. Cancers.

[15] Viviani S., Caccavari V., Gerardi C., Allocati E., Minoia C., Di Russo A. (2021). Male and female fertility: Prevention and monitoring in classical Hodgkin lymphoma and diffuse large B cell Lymphoma survivors. A systematic review by the Fondazione ItalianaLinfomi. Cancers.

[16] Daniele A., Guarini A., Summa S., Dellino M., Lerario G., Ciavarella S., Ditonno P., Paradiso A.V., Divella R., Casamassima P. (2021). Body Composition Change, Unhealthy Lifestyles and Steroid Treatment as Predictor of Metabolic Risk in Non-Hodgkin’s Lymphoma Survivors. J. Pers. Med..

[17] Kreuser E.-D., Felsenberg D., Behles C., Seibt-Jung H., Mielcarek M., Diehl V., Dahmen E., Thiel E. (1992). Long-term gonadal dysfunction and its impact on bone mineralization in patients following COPP/ABVD chemotherapy for Hodgkin’s disease. Ann. Oncol..

[18] Kiserud C.E., Seland M., Holte H., Fosså A., Fosså S.D., Bollerslev J., Bjøro T., Loge J.H. (2015). Fatigue in male lymphoma survivors differs between diagnostic groups and is associated with latent hypothyroidism. Acta Oncol..

[19] Gebauer J., Fick E.-M., Waldmann A., Langer T., Kreitschmann-Andermahr I., Lehnert H., Katalinic A., Brabant G. (2015). Self-reported endocrine late effects in adults treated for brain tumours, Hodgkin and non-Hodgkin lymphoma: A registry based study in Northern Germany. Eur. J. Endocrinol..

[20] Specht L., Yahalom J., Illidge T., Berthelsen A.K., Constine L.S., Eich H.T., Girinsky T., Hoppe R.T., Mauch P., Mikhaeel N.G. (2014). Modern Radiation Therapy for Hodgkin Lymphoma: Field and Dose Guidelines From theInternational Lymphoma Radiation Oncology Group (ILROG). Int. J. Radiat. Oncol. Biol. Phys..

[21] Yahalom J., Illidge T., Specht L., Hoppe R.T., Li Y.-X., Tsang R., Wirth A., International Lymphoma Radiation Oncology (2015). Modern Radiation Therapy for Extranodal Lymphomas: Field and Dose Guidelines from the International Lymphoma Radiation Oncology Group. Int. J. Radiat. Oncol. Biol. Phys..

[22] Moher D., Liberati A., Tetzlaff J., Altman D.G., The PRISMA Group (2009). Preferred Reporting Items for Systematic Reviews and Meta-Analyses: The PRISMA Statement. PLoS Med..

[23] Shea B.J., Reeves B.C., Wells G., Thuku M., Hamel C., Moran J., Moher D., Tugwell P., Welch V., Kristjansson E. (2017). AMSTAR 2: A critical appraisal tool for systematic reviews that include randomised or non-randomised studies of healthcare interventions, or both. BMJ.

[24] Higgins J.P., Altman D.G., Higgins J.P., Green S. (2010). The Cochrane Collaboration’s tool for assessing risk of bias. Cochrane Handbook for Systematic Reviews of Interventions.

[25] Wells G.S.B., O’connell D., Peterson J., Welch V., Losos M., Tugwell P. (1999). The Newcastle-Ottawa Scale (NOS) for Assessing the Quality of Non-randomised Studies in Meta-Analyses.

[26] MauriziEnrici R., Anselmo A.P., Donato V., FalchettoOsti M., Santoro M., Tombolini V., Mandelli F. (1999). Relapse and late complications in early-stage Hodgkin’s disease patients with mediastinal involvement treated with radiotherapy alone or plus one cycle of ABVD. Haematologica.

[27] Bethge W., Guggenberger D., Bamberg M., Kanz L., Bokemeyer C. (2000). Thyroid toxicity of treatment for Hodgkin’s disease. Ann. Hematol..

[28] Illés A., Bíró E., Miltényi Z., Keresztes K., Váróczy L., András C., Sipka S., Bakó G. (2003). Hypothyroidism and thyroiditis after therapy for Hodgkin’s disease. Acta Haematol..

[29] Pinnix C.C., Cella L., Andraos T.Y., Ayoub Z., Milgrom S.A., Gunther J., Thosani S., Wogan C., Conson M., D’Avino V. (2018). Predictors of Hypothyroidism in Hodgkin Lymphoma Survivors After Intensity Modulated Versus 3-Dimensional Radiation Therapy. Int. J. Radiat. Oncol. Biol. Phys..

[30] King D.J., Ratcliffe M.A., Dawson A.A., Bennett B., Macgregor J.E., Klopper A.I. (1985). Fertility in young men and women after treatment for lymphoma: A study of a population. J. Clin. Pathol..

[31] Meissner J., Tichy D., Katzke V., Kühn T., Dietrich S., Schmitt T., Ziepert M., Kuhnt E., Rixecker T., Zorn M. (2015). Long-term ovarian function in women treated with CHOP or CHOP plus etoposide for aggressive lymphoma. Ann. Oncol..

[32] Svendsen P., Shekhrajka N., Nielsen K.L., Vestergaard P., Poulsen M.Ø., Vistisen A.K., Munksgaard P.S., Severinsen M.T., Jensen P., Johnsen H.E. (2017). R-CHOP(-like) treatment of diffuse large B-cell lymphoma significantly reduces CT-assessed vertebral bone density: A single center study of 111 patients. Leuk. Lymphoma.

[33] Sklar C., Whitton J., Mertens A., Stovall M., Green D., Marina N., Greffe B., Wolden S., Robison L. (2000). Abnormalities of the thyroid in survivors of Hodgkin’s disease: Data from the Childhood Cancer Survivor Study. J. Clin. Endocrinol. Metab..

[34] Bhatia S., Ramsay N.K., Bantle J.P., Mertens A., Robison L.L. (1996). Thyroid abnormalities after therapy for Hodgkin’s disease in childhood. Oncologist.

[35] Majhail N.S., Ness K.K., Burns L.J., Sun C.-L., Carter A., Francisco L., Forman S.J., Bhatia S., Baker K.S. (2007). Late Effects in Survivors of Hodgkin and Non-Hodgkin Lymphoma Treated with Autologous Hematopoietic Cell Transplantation: A Report from the Bone Marrow Transplant Survivor Study. Biol. Blood Marrow Transplant..

[36] Flora E., van Leeuwen F., Ng A.K. (2016). Long-term risk of second malignancy and cardiovascular disease after Hodgkin lymphoma treatment. Hematology.

[37] Michaelson E.M., Chen Y.-H., Silver B., Tishler R.B., Marcus K.J., Stevenson M.A., Ng A.K. (2014). Thyroid Malignancies in Survivors of Hodgkin Lymphoma. Int. J. Radiat. Oncol. Biol. Phys..

[38] Chowdhry A.K., Fung C., Chowdhry V.K., Bergsma D., Dhakal S., Constine L.S., Milano M.T. (2017). A population-based study of prognosis and survival in patients with second primary thyroid cancer after Hodgkin lymphoma. Leuk. Lymphoma.

[39] Taylor A.J., Croft A.P., Palace A.M., Winter D.L., Reulen R.C., Stiller C.A., Stevens M.C.G., Hawkins M.M. (2009). Risk of thyroid cancer in survivors of childhood cancer Results from the British Childhood Cancer Survivor Study. Int. J. Cancer.

[40] Tonorezos E.S., Barnea D., Moskowitz C.S., Chou J.F., Sklar C.A., Elkin E.B., Wong R.J., Li D., Tuttle R.M., Korenstein D. (2017). Screening for Thyroid Cancer in Survivors of Childhood and Young Adult Cancer Treated with Neck Radiation. J. Cancer Surviv..

[41] Francis G.L., Waguespack S.G., Bauer A.J., Angelos P., Benvenga S., Cerutti J.M., Dinauer C.A., Hamilton J., Hay I.D., Luster M. (2015). Management Guidelines for Children with Thyroid Nodules and Differentiated Thyroid Cancer. Thyroid. Off. J. Am. Thyroid. Assoc..

[42] Gharib H., Papini E., Garber J.R., Duick D.S., Harrell R.M., Hegedus L., Paschke R., Valcavi R., Vitti P. (2016). American Association of Clinical Endocrinologists, American College of Endocrinology, and Associazione Medici Endocrinologi Medical Guidelines for Clinical Practice for the Diagnosis and Management of Thyroid Nodules—2016 Update Appendix. Endocr. Pract..

[43] Gharib H., Papini E., Garber J.R., Duick D.S., Harrell R.M., Hegedüs L., Paschke R., Valcavi R., Vitti P. (2016). Aace/ace/ame task force on thyroid nodules. American association of clinical endocrinologists, American college of endocrinology, and associazione medici endocrinologi medical guidelines for clinical practice for the diagnosis and management of thyroid nodules—2016 update. Endocr. Pract..

[44] Surks M.I., Ortiz E., Daniels G.H., Sawin C.T., Col N.F., Cobin R.H., Franklyn J.A., Hershman J.M., Burman K.D., Denke M.A. (2004). Subclinical thyroid disease: Scientific review and guidelines for diagnosis and management. JAMA.

[45] Garber J.R., Cobin R.H., Gharib H., Hennessey J.V., Klein I., Mechanick J.I., Pessah-Pollack R., Singer P.A., Woeber K.A. (2012). Clinical practice guidelines for hypothyroidism in adults: Cosponsored by the American Association of Clinical Endocrinologists and the American Thyroid Association. Endocr. Pract..

[46] Silvestris E., Cormio G., Skrypets T., Dellino M., Paradiso A.V., Guarini A., Minoia C. (2020). Novel aspects on gonadotoxicity and fertility preservation in lymphoproliferative neoplasms. Crit. Rev. Oncol. Hematol..

[47] Wallace W.H.B., Thomson A.B., Kelsey T.W. (2003). The radiosensitivity of the human oocyte. Hum. Reprod..

[48] Qu N., Itoh M., Sakabe K. (2019). Effects of Chemotherapy and Radiotherapy on Spermatogenesis: The Role of Testicular Immunology. Int. J. Mol. Sci..

[49] Bonadonna G., Santoro A., Viviani S., Lombardi C., Ragni G. (1984). Gonadal damage in Hodgkin’s disease from cancer chemotherapeutic regimens. Arch. Toxicol..

[50] Haukvik U., Dieset I., Bjøro T., Holte H., Fossa S.D. (2006). Treatment-related premature ovarian failure as a long-term complication after Hodgkin’s lymphoma. Ann. Oncol..

[51] Behringer K., Mueller H., Goergen H., Thielen I., Eibl A.D., Stumpf V., Wessels C., Wiehlpu¨tz M., Rosenbrock J., Halbsguth T. (2013). Gonadal Function and Fertility in Survivors After Hodgkin Lymphoma Treatment Within the German Hodgkin Study Group HD13 to HD15 Trials. J. Clin. Oncol..

[52] Bokemeyer C., Schmoll H.-J., van Rhee J., Kuczyk M., Schuppert F., Poliwoda H. (1994). Long-term gonadal toxicity after therapy for Hodgkin’s and non-Hodgkin’s lymphoma. Ann. Hematol..

[53] Kiserud C.E., Fossa A., Bjøro T., Holte H., Cvancarova M., Fossa S.D. (2009). Gonadal function in male patients after treatment for malignant lymphomas, with emphasis on chemotherapy. Br. J. Cancer.

[54] Gareer W., Gad Z., Gareer H. (2011). Needle oophoropexy: A new simple technique for ovarian transposition prior to pelvic irradiation. Surg. Endosc..

[55] Van Dorp W., Haupt R., Anderson R.A., Mulder R.L., van den Heuvel-Eibrink M.M., Broeder E.V.D.-D., Su H.I., Winther J.F., Hudson M.M., Levine J.M. (2018). Reproductive Function and Outcomes in Female Survivors of Childhood, Adolescent, and Young Adult Cancer: A Review. J. Clin. Oncol..

[56] Michalczyk K., Cymbaluk-Płoska A. (2021). Fertility Preservation and Long-Term Monitoring of Gonadotoxicity in Girls, Adolescents and Young Adults Undergoing Cancer Treatment. Cancers.

[57] Cabanillas M.E., Lu H., Fang S., Du X.L. (2007). Elderly patients with non-Hodgkin lymphoma who receive chemotherapy are at higher risk for osteoporosis and fractures. Leuk. Lymphoma..

[58] Holmes S.J., Whitehouse R.W., Clark S.T., Crowther D.C., Aams J.E., Shalet S.M. (1994). Reduced bone mineral density in men following chemotherapy for Hodgkin’s disease. Br. J. Cancer.

[59] Paccou J., Merlusca L., Henry-Desailly I., Parcelier A., Gruson B., Royer B., Charbonnier A., Ursu D., Desailloud R., Garidi R. (2014). Alterations in bone mineral density and bone turnover markers in newly diagnosed adults with lymphoma receiving chemotherapy: A 1-year prospective pilot study. Ann. Oncol..

[60] Buckley L., Guyatt G., Fink H.A., Cannon M., Grossman J., Hansen K.E., Humphrey M.B., Lane N.E., Magrey M., Miller M. (2017). American College of Rheumatology Guideline for the prevention and treatment of glucocorticoid-induced osteoporosis. Arthritis Rheumatol..

[61] Wei R.L., Jung B.C., Manzano W., Sehgal V., Klempner S.J., Lee S.P., Ramsinghani N.S., Lall C. (2016). Bone mineral density loss in thoracic and lumbar vertebrae following radiation for abdominal cancers. Radiother. Oncol..

[62] US. Preventive Services Task Force (2018). Screening for Osteoporosis to Prevent Fractures: Recommendation Statement. Am. Fam. Physician..

[63] Ratcliffe M., Lanham S.A., Reid D.M., Dawson A.A. (1992). Bone mineral density (BMD) in patients with lymphoma: The effect of chemotherapy, intermittent corticosteroids and premature menopause. Hematol. Oncol..

[64] American Medical Association (2001). Expert Panel on Detection, Evaluation Treatment of High Blood Cholesterol in Adults—Executive Summary of The Third Report of The National Cholesterol Education Program (NCEP) Expert Panel on Detection, Evaluation, And Treatment of High Blood Cholesterol In Adults (Adult Treatment Panel III). JAMA.

[65] Sheldon T. (2001). Cancer survival rates continue to rise in the Netherlands. BMJ.

[66] Carr M.C. (2003). The emergence of the metabolic syndrome with menopause. J. Clin. Endocrinol. Metab..

[67] Laaksonen D.E., Niskanen L., Punnonen K., Nyyssönen K., Tuomainen T.P., Valkonen V.P., Salonen R., Salonen J.T. (2004). Testosterone and sex hormone-binding globulin predict the metabolic syndrome and diabetes in middle-aged men. Diabetes Care.

[68] Basaria S., Dobs A.S. (2001). Hypogonadism and androgen replacement therapy in elderly men. Am. J. Med..

[69] Meacham L.R., Sklar C.A., Li S., Liu Q., Gimpel N., Yasui Y., Whitton J.A., Stovall M., Robison L.L., Oeffinger K.C. (2009). Diabetes mellitus in long-term survivors of childhood cancer. Increased risk associated with radiation therapy: A report for the childhood cancer survivor study. Arch. Intern. Med..

[70] De Vathaire F., El-Fayech C., Ben Ayed F.F., Haddy N., Guibout C., Winter D., Thomas-Teinturier C., Veres C., Jackson A., Pacquement H. (2012). Radiation dose to the pancreas and risk of diabetes mellitus in childhood cancer survivors: A retrospective cohort study. Lancet Oncol..

[71] Van Nimwegen F.A., Schaapveld M., Janus C.P., Krol A.D., Raemaekers J.M., Kremer L.C., Stovall M., Aleman B.M., Van Leeuwen F.E. (2014). Risk of Diabetes Mellitus in Long-Term Survivors of Hodgkin Lymphoma. J. Clin. Oncol..

[72] Tonorezos E.S., Hudson M.M., Edgar A.B., Kremer L.C., Sklar C.A., Wallace W.H.B., Oeffinger K.C. (2015). Screening and management of adverse endocrine outcomes in adult survivors of childhood and adolescent cancer Emily S Tonorezos. Lancet Diabetes Endocrinol..

[73] Gallagher D., DeLegge M. (2011). Body composition (sarcopenia) in obese patients: Implications for care in the intensive care unit. J. Parenter. Enter. Nutr..

[74] Cleasby M.E., Jamieson P.M., Atherton P.J. (2016). Insulin resistance and sarcopenia: Mechanistic links between common co-morbidities. J. Endocrinol..

[75] Batsis J.A., Villareal D.T. (2018). Sarcopenic obesity in older adults: Aetiology, epidemiology and treatment strategies. Nat. Rev. Endocrinol..

[76] Fischetti F., Greco G., Cataldi S., Minoia C., Loseto G., Guarini A. (2019). Effects of Physical Exercise Intervention on Psychological and Physical Fitness in Lymphoma Patients. Medicina.

[77] Minoia C., Ciavarella S., Lerario G., Daniele A., De Summa S., Napolitano M., Guarini A. (2018). Improvable Lifestyle Factors in Lymphoma Survivors. Acta Haematol..

