# Supplementary material for: Late Endocrine and Metabolic Sequelae and Long-Term Monitoring of Classical Hodgkin Lymphoma and Diffuse Large B-Cell Lymphoma Survivors: A Systematic Review by the Fondazione Italiana Linfomi
Source: Cancers (Basel). 2022 Mar 10;14(6):1439. doi: 10.3390/cancers14061439 (PMC8946842; doi:10.3390/cancers14061439)
Supplement: Supplementary file 1 [file cancers-14-01439-s001.zip › S1.docx]

| Supplementary mat.1: **Example of search strategy used in MedLine and adapted to search the other databases.** |
| --- |
| - PICO thyropaties incidence   MedLine, Embase, and Cochrane databases up to October 2020  **"**(hodgkin disease) OR "Hodgkin Disease"[Mesh] OR (hodgkin lymphoma) OR (“diffuse large b cell lymphoma”) OR "Lymphoma, Large B-Cell, Diffuse"[Mesh] OR “DBLC[TIAB]” AND "surviv*" OR “long term survivor” OR “survivorship” OR cancer survivors[Mesh] AND ("Consolidation Chemotherapy"[Mesh] OR "Induction Chemotherapy" [Mesh]) OR "Radiotherapy"[Mesh] OR “conventional chemotherapy” OR “chemotherap*” OR “treatment” OR “ABVD” OR “doxorubicin bleomycin vinblastine dacarbazine” OR “RCHOP” OR “CHOP” OR “rituximab cyclophosphamide doxorubicin vincristine prednisone” OR “high dose chemotherapy” OR “autologous stem cell transplant” OR “transplantation” OR “transplant” OR “haematopoietic stem cell graft” OR “brentuximab” OR “Antineoplastic Agents” [MH] OR "Antineoplastic Protocols"[Mesh] OR "Chemoradiotherapy"[Mesh] OR "Chemotherapy, Adjuvant"[Mesh] OR "Antineoplastic Combined Chemotherapy Protocols"[Mesh] AND “hypothyroidism” OR “hyperthyroidism” OR “autoimmune thyroid disease” OR “thyroiditis” OR “Hashimoto’s disease” OR “Graves’ disease” OR “nodule” OR “goiter” OR “thyroid cancer” OR “papillary” OR “follicular” OR Thyroid Diseases[Mesh] OR Carcinoma, Papillary, Follicular[Mesh] |
| - PICO gonadic dysfunction incidence   MedLine, Embase, and Cochrane databases up to October 2020  (hodgkin disease) OR "Hodgkin Disease"[Mesh] OR (hodgkin lymphoma) OR (“diffuse large b cell lymphoma”) OR "Lymphoma, Large B-Cell, Diffuse"[Mesh] OR “DBLC[TIAB]” AND "surviv*" OR “long term survivor” OR “survivorship” OR cancer survivors[Mesh] AND ("Consolidation Chemotherapy"[Mesh] OR "Induction Chemotherapy" [Mesh]) OR "Radiotherapy"[Mesh] OR “conventional chemotherapy” OR “chemotherap*” OR “treatment” OR “ABVD” OR “doxorubicin bleomycin vinblastine dacarbazine” OR “RCHOP” OR “CHOP” OR “rituximab cyclophosphamide doxorubicin vincristine prednisone” OR “high dose chemotherapy” OR “autologous stem cell transplant” OR “transplantation” OR “transplant” OR “haematopoietic stem cell graft” OR “brentuximab” OR “Antineoplastic Agents” [MH] OR "Antineoplastic Protocols"[Mesh] OR "Chemoradiotherapy"[Mesh] OR "Chemotherapy, Adjuvant"[Mesh] OR "Antineoplastic Combined Chemotherapy Protocols"[Mesh] AND “hypogonadism” OR “azoospermia” OR “oligospermia” OR“ testosterone” OR “infertility” OR “abnormal spermogram” “amenorrhea” OR “Vaginal dryness” OR “Hot flashes” OR“abnormal menses” OR “irregular menstrual cycles” OR “irregular periods” OR Gonadal Disorders[Mesh] OR Infertility[Mesh] OR Menstruation Disturbances[Mesh] OR Hot Flashes[Mesh] |
| - PICO bone disease incidence   MedLine, Embase, and Cochrane databases up to October 2020  (hodgkin disease) OR "Hodgkin Disease"[Mesh] OR (hodgkin lymphoma) OR (“diffuse large b cell lymphoma”) OR "Lymphoma, Large B-Cell, Diffuse"[Mesh] OR “DBLC[TIAB]” AND "surviv*" OR “long term survivor” OR “survivorship” OR cancer survivors[Mesh] AND ("Consolidation Chemotherapy"[Mesh] OR "Induction Chemotherapy" [Mesh]) OR "Radiotherapy"[Mesh] OR “conventional chemotherapy” OR “chemotherap*” OR “treatment” OR “ABVD” OR “doxorubicin bleomycin vinblastine dacarbazine” OR “RCHOP” OR “CHOP” OR “rituximab cyclophosphamide doxorubicin vincristine prednisone” OR “high dose chemotherapy” OR “autologous stem cell transplant” OR “transplantation” OR “transplant” OR “haematopoietic stem cell graft” OR “brentuximab” OR “Antineoplastic Agents” [MH] OR "Antineoplastic Protocols"[Mesh] OR "Chemoradiotherapy"[Mesh] OR "Chemotherapy, Adjuvant"[Mesh] OR "Antineoplastic Combined Chemotherapy Protocols"[Mesh] AND “osteoporosis” OR “osteopenia” OR “fracture risk” OR “fracture” OR Bone Diseases, Metabolic[Mesh] OR Fractures, Bone[Mesh] |
| - PICO therapy comparison (thyroid)   MedLine, Embase, and Cochrane databases up to January 2020  **(hodgkin disease) OR "Hodgkin Disease"[Mesh] OR (hodgkin lymphoma) OR (“diffuse large b cell lymphoma”) OR "Lymphoma, Large B-Cell, Diffuse"[Mesh] OR “DBLC[TIAB]”** AND "surviv*" OR “long term survivor” OR “survivorship” OR cancer survivors[Mesh] AND ("Consolidation Chemotherapy"[Mesh] OR "Induction Chemotherapy" [Mesh]) OR "Radiotherapy"[Mesh] OR “conventional chemotherapy” OR “chemotherap*” OR “treatment” OR “ABVD” OR “doxorubicin bleomycin vinblastine dacarbazine” OR “RCHOP” OR “CHOP” OR “rituximab cyclophosphamide doxorubicin vincristine prednisone” OR “high dose chemotherapy” OR “autologous stem cell transplant” OR “transplantation” OR “transplant” OR “haematopoietic stem cell graft” OR “brentuximab” OR “Antineoplastic Agents” [MH] OR "Antineoplastic Protocols"[Mesh] OR "Chemoradiotherapy"[Mesh] OR "Chemotherapy, Adjuvant"[Mesh] OR "Antineoplastic Combined Chemotherapy Protocols"[Mesh] OR Radiotherapy, Conformal"[Mesh] OR "Radiotherapy Planning, Computer-Assisted"[Mesh] OR (conformal radiotherapy) OR (3-D radiotherapy) OR (Intensity modulated radiotherapy) OR (Image-guided radiotherapy) OR (Proton therapy) OR (involved Nodal Radiotherapy) OR ( Involved Site Radiotherapy) OR ( Involved Field Radiotherapy) AND “hypothyroidism” OR “hyperthyroidism” OR “autoimmune thyroid disease” OR “thyroiditis” OR “Hashimoto’s disease” OR “Graves’ disease” OR “nodule” OR “goiter” OR “thyroid cancer” OR “papillary” OR “follicular” OR Thyroid Diseases[Mesh] OR Carcinoma, Papillary, Follicular[Mesh] |
| - PICO therapy comparison (gonads)   MedLine, Embase, and Cochrane databases  **(hodgkin disease) OR "Hodgkin Disease"[Mesh] OR (hodgkin lymphoma) OR (“diffuse large b cell lymphoma”) OR "Lymphoma, Large B-Cell, Diffuse"[Mesh] OR “DBLC[TIAB]”** AND "surviv*" OR “long term survivor” OR “survivorship” OR cancer survivors[Mesh] AND ("Consolidation Chemotherapy"[Mesh] OR "Induction Chemotherapy" [Mesh]) OR "Radiotherapy"[Mesh] OR “conventional chemotherapy” OR “chemotherap*” OR “treatment” OR “ABVD” OR “doxorubicin bleomycin vinblastine dacarbazine” OR “RCHOP” OR “CHOP” OR “rituximab cyclophosphamide doxorubicin vincristine prednisone” OR “high dose chemotherapy” OR “autologous stem cell transplant” OR “transplantation” OR “transplant” OR “haematopoietic stem cell graft” OR “brentuximab” OR “Antineoplastic Agents” [MH] OR "Antineoplastic Protocols"[Mesh] OR "Chemoradiotherapy"[Mesh] OR "Chemotherapy, Adjuvant"[Mesh] OR "Antineoplastic Combined Chemotherapy Protocols"[Mesh] OR Radiotherapy, Conformal"[Mesh] OR "Radiotherapy Planning, Computer-Assisted"[Mesh] OR (conformal radiotherapy) OR (3-D radiotherapy) OR (Intensity modulated radiotherapy) OR (Image-guided radiotherapy) OR (Proton therapy) OR (involved Nodal Radiotherapy) OR ( Involved Site Radiotherapy) OR ( Involved Field Radiotherapy) AND “hypogonadism” OR “azoospermia” OR “oligospermia” OR“ testosterone” OR “infertility” OR “abnormal spermogram” OR “amenorrhea” OR “Vaginal dryness” OR “Hot flashes” OR“abnormal menses” OR “irregular menstrual cycles” OR “irregular periods” OR Gonadal Disorders[Mesh] OR Infertility[Mesh] OR Menstruation Disturbances[Mesh] OR Hot Flashes[Mesh] |
| - PICO therapy comparison (bone)   MedLine, Embase, and Cochrane databases up to January 2020  **(hodgkin disease) OR "Hodgkin Disease"[Mesh] OR (hodgkin lymphoma) OR (“diffuse large b cell lymphoma”) OR "Lymphoma, Large B-Cell, Diffuse"[Mesh] OR “DBLC[TIAB]”** AND "surviv*" OR “long term survivor” OR “survivorship” OR cancer survivors[Mesh] AND ("Consolidation Chemotherapy"[Mesh] OR "Induction Chemotherapy" [Mesh]) OR "Radiotherapy"[Mesh] OR “conventional chemotherapy” OR “chemotherap*” OR “treatment” OR “ABVD” OR “doxorubicin bleomycin vinblastine dacarbazine” OR “RCHOP” OR “CHOP” OR “rituximab cyclophosphamide doxorubicin vincristine prednisone” OR “high dose chemotherapy” OR “autologous stem cell transplant” OR “transplantation” OR “transplant” OR “haematopoietic stem cell graft” OR “brentuximab” OR “Antineoplastic Agents” [MH] OR "Antineoplastic Protocols"[Mesh] OR "Chemoradiotherapy"[Mesh] OR "Chemotherapy, Adjuvant"[Mesh] OR "Antineoplastic Combined Chemotherapy Protocols"[Mesh] OR Radiotherapy, Conformal"[Mesh] OR "Radiotherapy Planning, Computer-Assisted"[Mesh] OR (conformal radiotherapy) OR (3-D radiotherapy) OR (Intensity modulated radiotherapy) OR (Image-guided radiotherapy) OR (Proton therapy) OR (involved Nodal Radiotherapy) OR ( Involved Site Radiotherapy) OR ( Involved Field Radiotherapy) AND “osteoporosis” OR “osteopenia” OR “fracture risk” OR “fracture” OR Bone Diseases, Metabolic[Mesh] OR Fractures, Bone[Mesh] |
| - PICO thyroid follow up   MedLine, Embase, and Cochrane databases up to October 2020  **(hodgkin disease) OR "Hodgkin Disease"[Mesh] OR (hodgkin lymphoma) OR (“diffuse large b cell lymphoma”) OR "Lymphoma, Large B-Cell, Diffuse"[Mesh] OR “DBLC[TIAB]”** AND "surviv*" OR “long term survivor” OR “survivorship” OR cancer survivors[Mesh] AND “thyroid function test*” OR “TSH” OR “thyroid ultrasound” OR Thyroid Function Tests [Mesh] Thyroid Diseases[Mesh] OR Carcinoma, Papillary, Follicular[Mesh] OR Thyroid Hormones[Mesh] AND “follow-up” OR “late side effect” OR “late adverse effect” OR “late onset” OR “late diagnosis” OR "long-term care" OR “long term” OR "patient care management" OR "patient care planning" OR "continuity patient care" OR OR "health planning guidelines" OR “secondary prevention”[Mesh] OR “secondary prevention” OR"Aftercare"[Mesh] aftercare [tiab] OR "Secondary Care"[Mesh] OR "Secondary Care" OR "Continuity of Patient Care"[Mesh] OR "Patient Care Management"[Mesh] OR "Health Planning Guidelines"[Mesh] OR "follow up" OR "late side effect" OR "late adverse effect" OR OR "late adverse event" OR "late adverse event rates" OR "late adverse events" OR "late adverse health effects" OR "late adverse reaction" OR "late adverse reactions" OR "late adverse side effects" OR "late adverse toxicity" OR "patient care plan" OR "patient care planning" OR "patient surveillance" OR surveillance[tiab] OR "patient monitoring" OR ((diagnosis[mh] OR diagnosis[sh] OR diagnos*[tiab]) AND (long OR late)) |
| - PICO gonads follow up   MedLine, Embase, and Cochrane databases up to October 2020  **(hodgkin disease) OR "Hodgkin Disease"[Mesh] OR (hodgkin lymphoma) OR (“diffuse large b cell lymphoma”) OR "Lymphoma, Large B-Cell, Diffuse"[Mesh] OR “DBLC[TIAB]”** AND "surviv*" OR “long term survivor” OR “survivorship” OR cancer survivors[Mesh] AND “testosterone” OR “LH” OR “LUTEINIZING HORMONE” OR “spermiogram” OR “sperm test” OR “FSH” OR “FOLLICLE STIMULATING HORMONE” OR “Inhibin B” OR “pelvic ultrasound” OR Gonadal Hormones[Mesh] OR Gonadotropin-Releasing Hormone[Mesh] OR Follicle Stimulating Hormone [Mesh] OR Luteinizing Hormone [Mesh] AND “follow-up” OR “late side effect” OR “late adverse effect” OR “late onset” OR “late diagnosis” OR "long-term care" OR “long term” OR "patient care management" OR "patient care planning" OR "continuity patient care" OR OR "health planning guidelines" OR “secondary prevention”[Mesh] OR “secondary prevention” OR"Aftercare"[Mesh] aftercare [tiab] OR "Secondary Care"[Mesh] OR "Secondary Care" OR "Continuity of Patient Care"[Mesh] OR "Patient Care Management"[Mesh] OR "Health Planning Guidelines"[Mesh] OR "follow up" OR "late side effect" OR "late adverse effect" OR OR "late adverse event" OR "late adverse event rates" OR "late adverse events" OR "late adverse health effects" OR "late adverse reaction" OR "late adverse reactions" OR "late adverse side effects" OR "late adverse toxicity" OR "patient care plan" OR "patient care planning" OR "patient surveillance" OR surveillance[tiab] OR "patient monitoring" OR ((diagnosis[mh] OR diagnosis[sh] OR diagnos*[tiab]) AND (long OR late)) |
| - PICO bone follow up   MedLine, Embase, and Cochrane databases up to October 2020  **(hodgkin disease) OR "Hodgkin Disease"[Mesh] OR (hodgkin lymphoma) OR (“diffuse large b cell lymphoma”) OR "Lymphoma, Large B-Cell, Diffuse"[Mesh] OR “DBLC[TIAB]”** AND "surviv*" OR “long term survivor” OR “survivorship” OR cancer survivors[Mesh] AND “serum calcium” OR “vitamin D” OR “bone mineral density” OR “DEXA scan” OR “DXA scan” OR Bone Diseases, Metabolic[Mesh] OR Fractures, Bone[Mesh] AND “follow-up” OR “late side effect” OR “late adverse effect” OR “late onset” OR “late diagnosis” OR "long-term care" OR “long term” OR "patient care management" OR "patient care planning" OR "continuity patient care" OR OR "health planning guidelines" OR “secondary prevention”[Mesh] OR “secondary prevention” OR"Aftercare"[Mesh] aftercare [tiab] OR "Secondary Care"[Mesh] OR "Secondary Care" OR "Continuity of Patient Care"[Mesh] OR "Patient Care Management"[Mesh] OR "Health Planning Guidelines"[Mesh] OR "follow up" OR "late side effect" OR "late adverse effect" OR OR "late adverse event" OR "late adverse event rates" OR "late adverse events" OR "late adverse health effects" OR "late adverse reaction" OR "late adverse reactions" OR "late adverse side effects" OR "late adverse toxicity" OR "patient care plan" OR "patient care planning" OR "patient surveillance" OR surveillance[tiab] OR "patient monitoring" OR ((diagnosis[mh] OR diagnosis[sh] OR diagnos*[tiab]) AND (long OR late)) |
| - PICO metabolic syndrome prevalence   MedLine, Embase, and Cochrane databases up to October 2019  ("epidemiology OR "prevalence"[All Fields] OR "prevalence"[MeSH Terms]) AND (((((((("Lymphoma, Large B-Cell, Diffuse"[Mesh]) OR "diffuse large b cell lymphoma") OR "hodgkin lymphoma") OR "Hodgkin Disease"[Mesh]) OR "hodgkin disease")) AND (("survivor" OR " long term survivor" OR "survivorship" OR cancer survivors OR “long-term lymphoma” OR “ adult lymphoma survivors”[Mesh]))) AND ("metabolic syndrome") [Mesh]) OR "metabolic " OR “syndrome” [Mesh]) OR " metabolic syndrome and its components” AND “dyslipidemia and diabetes” [Mesh]) OR " ("diabetes mellitus"[MeSH Terms] OR ("diabetes"[All Fields] AND "mellitus"[All Fields]) OR "diabetes mellitus"[All Fields] OR "diabetes"[All Fields] OR "diabetes insipidus"[MeSH Terms] OR ("diabetes"[All Fields] AND "insipidus"[All Fields]) OR "diabetes insipidus"[All Fields]) AND ("dyslipidemias"[MeSH Terms] OR "dyslipidemias"[All Fields] OR "dyslipidemia"[All Fields]) AND ("obesity"[MeSH Terms] OR "obesity"[All Fields]) AND ("metabolic syndrome"[MeSH Terms] OR ("metabolic"[All Fields] OR "syndrome"[All Fields]) OR "metabolic syndrome"[All Fields]) AND ("sarcopenia"[MeSH Terms] OR "sarcopenia"[All Fields]) OR OR " loss lean muscle" (decrease AND lean[All Fields] AND ("muscles"[MeSH Terms] OR "muscles"[All Fields] OR "muscle"[All Fields])) nutrizional[All Fields] AND status[All Fields] AND FFM[All Fields] AND ("Financ Manage"[Journal] OR "Field methods"[Journal] OR "fm"[All Fields]) |
